# Autophagy as an Emerging Common Pathomechanism in Inherited Peripheral Neuropathies

**DOI:** 10.3389/fnmol.2017.00143

**Published:** 2017-05-11

**Authors:** Mansour Haidar, Vincent Timmerman

**Affiliations:** Peripheral Neuropathy Research Group, Institute Born Bunge, University of AntwerpAntwerpen, Belgium

**Keywords:** autophagy, hereditary neuropathies, Charcot-Marie-Tooth, neurodegeneration, proteostasis

## Abstract

The inherited peripheral neuropathies (IPNs) comprise a growing list of genetically heterogeneous diseases. With mutations in more than 80 genes being reported to cause IPNs, a wide spectrum of functional consequences is expected to follow this genotypic diversity. Hence, the search for a common pathomechanism among the different phenotypes has become the holy grail of functional research into IPNs. During the last decade, studies on several affected genes have shown a direct and/or indirect correlation with autophagy. Autophagy, a cellular homeostatic process, is required for the removal of cell aggregates, long-lived proteins and dead organelles from the cell in double-membraned vesicles destined for the lysosomes. As an evolutionarily highly conserved process, autophagy is essential for the survival and proper functioning of the cell. Recently, neuronal cells have been shown to be particularly vulnerable to disruption of the autophagic pathway. Furthermore, autophagy has been shown to be affected in various common neurodegenerative diseases of both the central and the peripheral nervous system including Alzheimer’s, Parkinson’s, and Huntington’s diseases. In this review we provide an overview of the genes involved in hereditary neuropathies which are linked to autophagy and we propose the disruption of the autophagic flux as an emerging common pathomechanism. We also shed light on the different steps of the autophagy pathway linked to these genes. Finally, we review the concept of autophagy being a therapeutic target in IPNs, and the possibilities and challenges of this pathway-specific targeting.

## Introduction

Disruption of intracellular homeostasis is at the base of most pathological conditions. Cellular health and function often relies on the maintenance of protein homeostasis by assuring that structurally abnormal proteins do not accumulate in cells, causing aggregate formation and organelle damage. The function of proteostasis is accomplished by two systems, the proteasome and the autophagy system. While the proteasome targets ubiquitinated and short-lived proteins, autophagy can degrade long-lived proteins and damaged organelles. Three main subtypes of autophagy have been identified, microautophagy, CMA, and macroautophagy. Microautophagy involves the degradation of cytosolic material inside the lysosome by direct lysosomal invagination (Mijaljica et al., 2011). CMA is a specific autophagic pathway where chaperones target proteins containing the KFERQ motif to the lysosome for degradation (Cuervo et al., 1995). Unlike CMA, macroautophagy (the main focus of this review), is a bulk degradation process characterized by the formation of double membrane vesicles, autophagosomes, which engulf cytoplasmic material and degrade their contents by fusing with the lysosomes (Yang and Klionsky, 2010). Disruption of the macroautophagy pathway can lead to failure to clear out misfolded proteins and dead organelles, or increased accumulation of autophagic structures and has been linked to neurodegeneration (Frake et al., 2015). Mutations in key autophagy regulating genes have been shown to cause neurodegeneration (Saitsu et al., 2013; Kyöstilä et al., 2015). Furthermore, mouse models with neuron-specific KO of autophagy genes frequently develop neurodegeneration (Komatsu et al., 2006). Interestingly, some of these mouse models display features of peripheral neuropathy as evident by decline in motor performance on the rota-rod, limb-clasping, and paw placement tests (Hara et al., 2006). The link between disruption at the gene level and the development of peripheral neuropathy is the main feature of IPNs. IPNs are a genetically heterogeneous disease population with over 80 affected genes discovered so far (Baets et al., 2014; Timmerman et al., 2014). While the clinical presentation of IPN patients is rather common, with length-dependent degeneration affecting the motor and/or sensory nerves, the variety of associated genes has produced various molecular phenotypes (Pareyson et al., 2014; Weis et al., 2016). Recently, more and more studies have indicated the involvement of autophagic impairment in IPN causing-mutations, and important roles for IPN related genes in autophagy. While the evidence linking IPN associated genes to autophagy can be direct or indirect, impairment of autophagy presents as an important contributor to the neuropathic phenotype given the vulnerability of neurons, especially those of the peripheral nervous system to disrupted cellular recycling and clearance. Identifying a common pathomechanism among the different IPN-related genes would provide a great therapeutic potential for targeting these neuropathies. This review discusses the advances made so far regarding the cellular and molecular mechanisms behind the different forms of IPN due to impairment of the autophagic pathway.

## Autophagy

**FIGURE 1:**
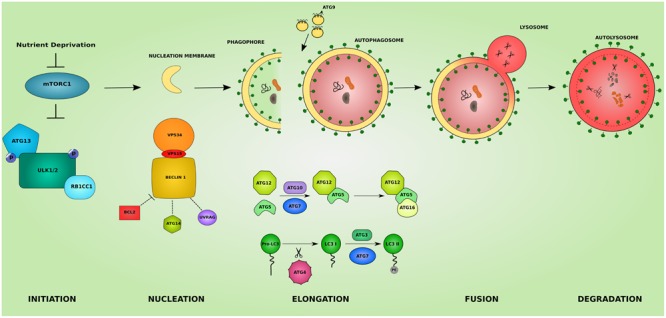
**The different steps of the autophagic pathway.** The autophagic pathway consists of several steps. The initiation step is governed by the initiation complex composed of ULK1 and ULK2 (*unc-51 like autophagy activating kinase 1/2*), RB1CC1 (*RB1 inducible coiled-coil 1*) and ATG13 and under the control of the nutrient sensing *mechanistic target of rapamaycin* (MTORC1) complex. After the initiation step, the nucleation of early autophagic membranes is controlled by the nucleation complex consisting of *phosphatidylinositol 3-kinase catalytic subunit type 3* (PIK3C3/VPS34), *beclin 1* (BECN1), and *phosphoinositide-3-kinase regulatory subunit 4* (PIK3R4/VPS15). BECN1 acts as an autophagy check point by interacting with ATG14, UVRAG (*UV radiation resistance associated*), and apoptosis regulator BCL2 proteins. The formed phagophore then undergoes elongation to become a fully closed double-membraned autophagosome. This step involves 2 conjugation systems resulting in the formation of ATG12-ATG5-ATG16L1 complex via ATG7 and ATG10 action, and of the autophagosomal marker LC3II via the action of ATG4, ATG7, and ATG3. The delivery of membranes to forming autophagosomes is served by ATG9-containing vesicles. The completed autophagosome then fuses with a lysosome becoming an autolysosome where its cargo is subjected to lysosomal degradation. The colors and shapes of the boxes are randomly assigned and show that they are different proteins belonging to the same complexes.

Macroautophagy, hereafter referred to as autophagy, is a homeostatic cellular process by which protein aggregates and cellular organelles are targeted, degraded, and recycled (**Figure 1**). Autophagy is a multi-step process consisting of: induction, nucleation of the isolation membrane (phagophore), elongation and expansion of the phagophore into a closed double-membraned autophagosome, lysosomal docking and fusion, and degradation of autophagic cargo. The different autophagy steps, explained below, are governed by a wide array of proteins and protein complexes, most notably the group of proteins encoded by the autophagy-related (*ATG*) genes (Parzych and Klionsky, 2014).

### Induction of Autophagy

The initiation of autophagy is regulated by the ULK complex composed of ULK1 and ULK2, and their stable interactors RB1CC1 and ATG13. The autophagy initiation complex is under the control of the nutrient-sensing, MTORC1, a serine–threonine kinase. During nutrient rich conditions MTORC1 binds to and phosphorylates ULK1/2 inhibiting their kinase activity. Removal of MTORC1 from the ULK1/2 complex, by nutrient starvation for example, allows for the activation of ULK1/2 and the proceeding of the autophagic process (Hosokawa et al., 2009).

### Phagophore Nucleation

The membrane nucleation step involves the conversion of PtdIns to phosphatidylinositol 3-phosphate (PtdIns3P) by the kinase complex Class III PtdIns3K. This complex consists of PIK3C3/VPS34, BECN1, and PIK3R4/VPS15 (Petiot et al., 2000). PtdIns3P is a signal for recruiting ATG proteins in the nucleation site, by recognizing and binding to PtdIns3P. BECN1 moderates the coupling of the PtdIns3K complex with various proteins, and via its binding partners serves as a check point that can inhibit or upregulate autophagy. Among the binding partners are ATG14, UVRAG, and apoptosis regulator BCL2 proteins. BCL2 binding suppresses autophagy (Pattingre et al., 2005). UVRAG binding targets the nucleation machinery to the endosomal membranes or the cell surface and can up or downregulate autophagy depending on its other binding partners (Yang and Klionsky, 2010). ATG14 binding on the other hand targets the nucleation to the omegasome (an ER resident precursor of the autophagosome where formation of autophagic vacuoles ensues) (Axe et al., 2008).

### Elongation and Expansion

Two ubiquitin-like conjugation systems are at the heart of the expansion of the phagophore into an autophagosome. The first system concerns the formation of ATG12-ATG5-ATG16L1 complex. ATG12 is covalently conjugated to ATG5 via the E1 activating enzyme ATG7 (Tanida et al., 1999) and the E2 conjugating enzyme ATG10 (Shintani et al., 1999). The ATG12-ATG5 complex then binds ATG16L1 through ATG5. The second ubiquitin-like conjugation reaction involves the lipidation of ATG8 (LC3) to ATG8-Phospatidylenolamine (LC3II). This first requires the C-terminal cleavage of LC3 by the protease ATG4. The cleaved protein (LC3I) is then processed by the E1 activating enzyme ATG7 and the E2 conjugating enzyme ATG3 yielding LC3II. LC3II remains associated with the phagophore and the mature autophagosomes until its degradation in the lysosome (Kabeya, 2000). This association makes LC3II a good marker for the study of the autophagic activity in cell and animal models. Since lipid synthesis does not occur at the phagophore, delivery of membrane from other locations in the cell is necessary for the elongation step. The task of membrane recruitment is served by ATG9, the only transmembrane protein in the ATG family. Mainly localized to the *trans*-Golgi network and the late endosomes, autophagy activation drives the trafficking of membrane delivering-ATG9 to the sites of autophagosome formation (Mari et al., 2010). Lipid delivery supplied by ATG9 allow for the elongation of the phagophore into a fully closed autophagosome.

### Fusion

The complete autophagosome eventually moves to and fuses with a lysosome becoming an autolysosome and/or with an endosome forming an amphisome. The transport of autophagosomes to the lysosomes depends on microtubules. The mechanism of docking at and fusion with the lysosome is not well-understood (Chen and Klionsky, 2011), but it is thought to involve UVRAG, RAB7 GTPase and syntaxin-17 (STX17) of the SNARE machinery (Jäger et al., 2004; Liang et al., 2008; Jiang et al., 2014).

### Degradation

Upon the fusion of the autophagosome with the lysosome, the autophagic cargo is degraded by various lysosomal hydrolases and proteases. The degradation products including metabolites, amino acids and fatty acids are recovered and reutilized by the cell.

### Autophagy in Neurons

The morphological features that distinguish neurons from other cells are their post-mitotic nature, highly polarized structure, and extended cytoplasm into axons and dendrites that can stretch far from the cell body. The latter feature is more exaggerated in neurons supplying the peripheral nerves. The spatial compartmentalization of neurons makes them prone to aggregation and accumulation of dead organelles and misfolded proteins. Autophagy therefore forms an essential homeostatic process for neurons. Knocking out key autophagy genes, such as ATG7 in mouse neurons leads to neurodegeneration (Komatsu et al., 2006). Defective autophagy has also been linked with numerous neurodegenerative diseases (Frake et al., 2015). The high susceptibility of neurons to autophagic impairment could explain why mutations in ubiquitously expressed genes can cause neuron-specific pathology in inherited neuropathies.

## Inherited Peripheral Neuropathies (IPNs)

Inherited peripheral neuropathies are a genetically heterogeneous group of disorders affecting the peripheral nerves. Depending on the affected nerves, IPNs are classified as CMT or HMSN if motor and sensory nerves are affected. If sensory and/or autonomic nerve dysfunction predominates, the neuropathy is termed (HSAN) and dHMN if motor deficits are the most prevalent (Pareyson et al., 2014).

### Clinical and Inheritance Pattern Classification

Charcot-Marie-Tooth, the most common form of hereditary neuropathies affects about 1:2500. Patients with CMT share a common clinical presentation of slowly progressive muscle wasting and weakness ascending from the feet to reach the thighs and hands, reduced tendon reflexes, skeletal deformities, and sometimes sensory loss (Harding and Thomas, 1980b). In many cases, the age of onset is in the first to second decade, but infantile, early-onset and late-onset forms exist. CMT is further classified on the prevalence of myelin or axonal involvement and according to the mode of inheritance. CMT1, the demyelinating subgroup is associated with reduced NCVs to less than 35 m/s. CMT2, the axonal subgroup on the other hand, only shows slightly reduced NCV but with reduced CMAPs amplitudes. An intermediate CMT subgroup exists with NCV between 35 and 45 m/s. The mode of inheritance further subdivides CMT neuropathies into CMT4 for recessive demyelinating CMT, CMT2R for recessive axonal CMT, CMTDI, and CMTRI for dominant and recessive intermediate CMT respectively, and CMT-X for the X-linked CMT (Stojkovic, 2016).

The **HSAN** disorders are characterized by sensory deficits and the involvement of the autonomic dysfunction. Five types of HSAN are acknowledged depending on the inheritance pattern and the sensory abnormality (Thomas, 2005). HSAN type I is autosomal dominant with variable motor involvement. HSAN types II to VI are autosomal recessive and further classified according to the sensory involvement and the clinical presentation ranging from osteomyelitis in type II to respiratory difficulties in type VI. HSAN types III, IV, and V show congenital onset. HSAN with spastic paraplegia, also a recessive form of HSAN, presents as a sensory neuropathy with mild spastic paraplegia (Rotthier et al., 2012).

The **dHMN** are classified into seven subtypes depending on the age of onset, inheritance pattern, and the distribution of the deficits (Harding and Thomas, 1980a). The dHMN types I, II, V, and VII are autosomal dominant, while types III, IV, and VI are autosomal recessive and the X-linked dHMN has an X-linked inheritance. Minor sensory abnormalities are frequent to many dHMN forms, and an overlap with axonal CMT (CMT2), where mutations in the same gene can cause either phenotypes is common. The phenotypic characteristics of dHMN are very diverse with the pathology involving other organs such as the vocal cords or the respiratory system (Rossor et al., 2012).

## Gene Function Classification

**FIGURE 2:**
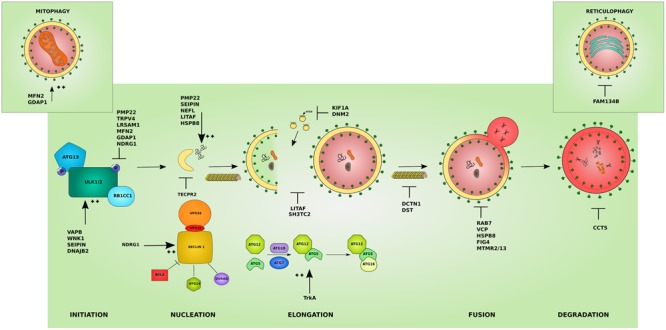
**Effects of the different IPN-associated genes on the autophagy pathway.** Inherited peripheral neuropathy associated genes disrupt autophagy at various levels. Several affected proteins disrupt the initiation of autophagy by inhibiting the initiation complex these include: *peripheral myelin protein 22* (PMP22), involved in more than 50% of IPNs, *transient receptor potential cation channel subfamily V member 4* (TRPV4), and *leucine rich repeat and sterile alpha motif containing 1* (LRSAM1). Others overstimulate the initiation step such as: *VAMP associated protein B* (VAPB), *WNK lysine deficient protein kinase 1* (WNK1), *seipin lipid droplet biogenesis associated* (Seipin), and *DnaJ heat shock protein family (Hsp40) member B2* (DNAJB2). Mutations affecting mitochondrial proteins can inhibit autophagy by causing an abnormal increase in mitophagy and disrupting the autophagy/mitophagy balance: *mitofusin 2* (MFN2) and *ganglioside induced differentiation associated protein 1* (GDAP1) (left inset). Mutant ER-resident *family with sequence similarity 134 member B* (FAM134B) affects its role in reticulophagy (right inset). The *cyto-protective N-myc downstream regulated 1* (NDRG1) inhibits the initiation complex or stimulates the nucleation complex depending on the physiological triggers. At the nucleation step, mutant *tectonin beta-propeller repeat containing 2* (TECPR2) disrupts the formation of early autophagic membranes from the ER. The elongation of the phagophore into an autophagosome is disrupted by mutations that affect the supply for forming membranes from late endosomes: *lipopolysaccharide induced TNF factor* (LITAF) and *SH3 domain and tetratricopeptide repeats 2* (SH3TC2), and from ATG9-containing vesicles as in the microtubules associated proteins: *kinesin family member 1A* (KIF1A) and *Dynamin 2* (DNM2). This step can also be overstimulated by mutations in the *tyrosine kinase A* (TrkA) which lead to toxic increase in ATG12-ATG5 conjugates. The transport of autophagosomes to lysosomes is disrupted by mutations affecting cytoskeleton associated proteins such as *Dynactin 1* (DCTN1) and *dystonin* (DST). Mutations involving *Ras-related GTPase* (RAB7), *valosin containing protein* (VCP), *heat shock protein B8* (HSPB8), and the phosphatases: *phosphoinositide 5-phosphatase* (FIG4), and *myotubularin-related* proteins (MTMR2 and MTMR13) block the lysosomal fusion step in autophagy. Mutant *chaperonin containing TCP1 subunit 5* (CCT5) on the other hand inhibits the degradation step. In addition, several mutant proteins lead to the formation of aggregates which basal autophagy on its own might not cope with (PMP22, Seipin, LITAF, HSPB8) and *neurofilament light* (NEFL). The colors and shapes of the boxes are randomly assigned and show that they are different proteins belonging to the same complexes.

Hereditary peripheral neuropathies show overlap on the genetic level with mutations in the same gene leading to different clinical subtypes. This has recently lead to attempts in classifying IPNs based on the gene function involved in neuropathy (Vallat et al., 2016). While many genes associated with neuropathy have a known function in neuronal maintenance, development, or myelination, several other genes revealed functions that were unrecognized or indirect to the caused neuropathy (Weis et al., 2016). The categorization based on gene function further highlighted the heterogeneity of inherited neuropathies. Hence, the search for a common pathomechanism among the different causal genes is starting to become a focal point in the hope of better understanding the pathology of inherited neuropathies and ultimately designing efficient therapeutic approaches. We believe that this classification is of great help for a better understanding of the molecular consequences of the disease-causative mutations. In the following sections, we shed light on the involvement of IPN-associated genes in the different steps of the autophagy pathway presenting autophagic impairment (**Figure 2**) as an emerging common pathomechanism (summarized in **Table 1**).

**Table 1:** Summary of IPN-associated genes, their clinical phenotype and the stage they impact the autophagy pathway.

Affected autophagy step/type	IPN-associated gene	Clinical phenotype
Initiation	*PMP22* (peripheral myelin protein 22)	CMT1A, CMT1E, HNPP
	*VAPB* (VAMP associated protein B)	Late onset SMA, ALS8
	*WNK1* (WNK lysine deficient protein kinase 1)	HSAN-IIA
	*BSCL2* (Seipin)	dHMN-V
	*LRSAM1* (leucine rich repeat and sterile alpha motif containing 1)	CMT2P
	*TRPV4* (transient receptor potential cation channel subfamily V member 4)	CMT2C, congenital distal SMA
	*NDRG1* (N-myc downstream regulated 1)	CMT4D
	*DNAJB2/HSJ1* [DnaJ heat shock protein family (Hsp40) member B2]	AR-dHMN
	*NEFL* (neurofilament light)	CMT2E, CMT1F
Autophagosome formation and expansion	*TrkA (NTRK1)* (neurotrophic receptor tyrosine kinase 1)	HSAN-IV, CIPA
	*DNM2* (dynamin2)	DI-CMTB
	*KIF1A* (kinesin family member 1A)	HSAN-IIC
	*LITAF* (lipopolysaccharide-induced TNF factor)	CMT1C
	*SH3TC2* (SH3 domain and tetratricopeptide repeats 2)	CMT4C
	*TECPR2* (tectonin beta-propeller repeat containing 2)	HSAN-III, HSP
Transport of autophagosomes	*DCTN1* (dynactin subunit 1)	dHMN-VIIb
	*DST* (dystonin)	HSAN-VI
Lysosomal fusion and degradation	*RAB7* (member RAS oncogene family)	CMT2B
	*CCT5* (chaperonin containing TCP-1 subunit 5)	HSNSP
	*FIG4* (FIG4 phosphoinositide 5-phosphatase)	CMT4J
	*MTMR2; MTMR13* (myotubularin-related proteins)	CMT4B1, CMT4B2
	*VCP* (valosin containing protein)	CMT2Y, ALS8, IBMPFD
	*HSPB8* (heat shock protein B8)	CMT2L, dHMN-I
Mitophagy	*MFN2* (mitofusin 2)	CMT2A
	*GDAP1* (ganglioside induced differentiation associated protein 1)	CMT4A, AR-CMT2, CMT2K
Reticulophagy	*FAM134B* (family with sequence similarity 134 member B)	HSAN-IIB

### IPN Genes and Autophagy Initiation

#### PMP22 (Peripheral Myelin Protein 22)

The PMP22 is a myelin glycoprotein which plays an important role in the formation and maintenance of compact myelin hence participating in the formation of the myelin sheath (Li et al., 2012). Autophagy is also involved in myelination of sciatic nerves by regulating the structural plasticity of Schwann cells (Jang et al., 2015). Mutations in *PMP22* are responsible for more than 50% of all inherited neuropathies. Duplication of *PMP22* causes the most common demyelinating neuropathy, CMT1A (Lupski et al., 1992; Timmerman et al., 1992). Point mutations in *PMP22* cause CMT1E neuropathy, while a heterozygous deletion cause hereditary neuropathy with liability to pressure palsies (HNPP) (Nicholson et al., 1994; Russo et al., 2011). CMT1A and CMT1E patients show variable clinical severity but the main features of the diseases include slowly progressive weakness and atrophy of the distal limb muscles, *pes cavus*, and reduced or absent deep tendon reflexes. In the *Trembler* CMT1E mouse models carrying a missense mutation in *Pmp22*, the mutant protein colocalizes with lysosomal markers (Notterpek et al., 1997). In the C22 mouse model of overexpression of PMP22, resembling the CMT1A phenotype, aggregate formation is seen in affected nerves. This aggregation occurs together with a reduced proteasomal activity and association of the aggregates with autophagosomes (Fortun et al., 2006). Since autophagy and the proteasome can play compensatory roles in maintaining cellular homeostasis, autophagy seems to play a part in the pathomechanism of CMT1 neuropathic mouse models. With reduced proteasomal activity, basal autophagy on its own might not be enough to clear out all the aggregating mutant proteins, but induction of autophagy by external methods can aid in the degradation of toxic products. Indeed, autophagy induction in neuronal cultures from CMT1A mouse models via nutrient deprivation or rapamycin treatment decreases the aggregate formation. Autophagy induction also improves PMP22 trafficking leading to an enhancement of remyelination and indirectly increasing the expression of myelin proteins and the abundance and length of myelin internodes (Madorsky et al., 2009; Rangaraju et al., 2010). Suppression of a key autophagy protein, ATG12 by siRNA, abolishes these effects, indicating that the phenotypic improvements are mediated by autophagy and that an intact autophagy pathway is required for proper remyelination (Rangaraju et al., 2010).

#### VAPB (VAMP Associated Proteins B and C)

VAMP associated proteins B and C belongs to a family of ER anchored proteins and plays a role in Golgi-mediated transport, membrane trafficking and neurotransmitter release (Lev et al., 2008). Recently the tethering complex formed by VAPB (ER) and PTPIP51 (mitochondria) has been shown to regulate the induction of autophagy through a role in mitochondria-ER calcium delivery which acts on BCL2-Beclin 1 interaction (Gomez-Suaga et al., 2017). Dominant mutations in *VAPB* lead to complex and atypical forms of inherited motor neuron disease (Nishimura et al., 2004). In a VAPB/ALS8 knock-in mouse model, mutant VAPB translocates from the ER to the autophagosome where it is degraded (Larroquette et al., 2015). The mislocalisation of VAPB causes ER stress and can lead to loss of VAPB functions in the ER. Absence of VAPB in the ER abolishes the VAPB-PTPIP51 tethering complex. Loosening of the ER-mitochondria contacts by loss of VAPB1-PTPIP51 can lead to overstimulation of autophagy (Gomez-Suaga et al., 2017). The dysregulated autophagy may have adverse effects on neuronal homeostasis and might be a prime pathological sign in VAPB associated neuropathies.

#### WNK1 (WNK Lysine Deficient Protein Kinase 1)

WNK lysine deficient protein kinase 1 is a serine/threonine kinase with the canonical function of regulating ion transport across cell membranes (Moriguchi et al., 2005). Truncating mutations in *WNK1* cause hereditary sensory and autonomic neuropathy type II (HSAN-II) (Rivière et al., 2004). HSAN-II is characterized by absence of pain sensations and patients suffer from ulcero-mutilating form of neuropathy. Little is known about the non-canonical functions of WNK1, but a role in autophagy was discovered recently. WNK1 exerts an inhibitory effect on basal and starvation-induced autophagy by interacting with UV Radiation Resistance-Associated Gene (UVRAG), a component of the main autophagy complex PI3KC3. WNK1 interaction reduces the activity of PI3KC3 and alters the phosphorylation status of ULK1 leading to inhibition of autophagy. Knocking-down WNK1 by siRNA in different cell lines leads to increased activation of autophagy (Gallolu Kankanamalage et al., 2016). WNK1 appears to play a regulatory role in autophagy by inhibiting the process, therefore truncating mutations in *WNK1* leading to neuropathy might hinder its autophagy-related function and lead to toxic increase in autophagic flux that can have deleterious effects contributing to the neuropathology.

#### BSCL2 (Seipin)

The *BSCL2* gene encodes the protein seipin, a transmembrane protein that resides in the ER (Ito et al., 2008). The exact function of seipin remains elusive, though a role in lipid homeostasis and adipogenesis has been suggested (Cartwright and Goodman, 2012). Null mutations in *BSCL2* are responsible of lipodystrophy, dominant mutations on the other hand cause distal hereditary motor neuropathy (dHMN-V) and Silver syndrome. dHMN-V patients present with uncharacteristic prominent hand muscle wasting and weakness early in the course of the disease, and mild to severe plasticity of the lower limbs (Windpassinger et al., 2004). Neuropathy-causing *BSCL2* mutations have been shown to affect the glycosylation sites of BSCL2 and lead to the accumulation of the unfolded protein in the ER (Windpassinger et al., 2004). It has been confirmed that different mutations in *BSCL2* can lead to the formation of perinuclear aggregates (Hsiao et al., 2016). In a neuropathic mouse model expressing mutant seipin exclusively in neurons (seipinopathy), seipin presents in intracellular aggregates. In addition, motor neurons show accumulation of autophagy marker LC3II together with a fragmented Golgi apparatus phenotype (Guo et al., 2013). The accumulation of autophagosomes was also confirmed by electron microscopy indicating that overstimulation of autophagy by mutant seipin leads to signs of degeneration (fragmented Golgi). The increased activation of autophagy was also confirmed in cell models expressing mutant seipin (Fan et al., 2015). Together these studies show that disrupted autophagy is a hallmark of seipin-related neuropathy.

#### LRSAM1 (Leucine Rich Repeat and Sterile Alpha Motif Containing 1)

Leucine rich repeat and sterile alpha motif containing 1 is a leucine-rich repeat protein and an E3 ubiquitin ligase. LRSAM1 is involved in cell adhesion and cargo sorting during receptor endocytosis (Amit et al., 2004). Dominant negative mutations in *LRSAM1* cause CMT2P neuropathy (Weterman et al., 2012). CMT2P patients display features of the axonal CMT2 including mild lower-limb sensorimotor neuropathy, foot deformities, and hammer toes (Weterman et al., 2012). Early studies reported a function for LRSAM1 in endocytosis and retrovirus budding (Amit et al., 2004). Later it was shown to recognize, ubiquitinate and guide several bacteria strains to autophagy (bacteriophagy) (Huett et al., 2012). LRSAM1 is also a potential interactor of LC3 family protein GAPARAPL2 (GABA type A receptor-associated protein) (Ng et al., 2011), and is also involved in the autophagy pathway via interaction with PHD finger protein 23 (PHF23), a negative regulator of autophagy (Wang et al., 2014). A role for LRSAM1 in the general autophagic pathway is evident by the fact that LRSAM1 overexpression increases autophagic flux by increasing the formation of LC3-GFP puncta in cultured cell lines, while silencing it causes a decrease in autophagic flux (Wang et al., 2014). LRSAM1 appears to play a regulatory role in the activation of autophagy probably through the interaction with other signaling proteins. Though the exact connection between LRSAM1, interactors and general autophagy remains unclear, loss of function mutations may disrupt the role of LRSAM1 in autophagy.

#### TRPV4 (Transient Receptor Potential Cation Channel Subfamily V Member 4)

Transient receptor potential cation channel subfamily V member 4 is a member of the TRP cation channels, and has an important role in sensing temperature, osmotic pressure, and mechanic stimuli (Köttgen et al., 2008). Mutations in *TRPV4* cause CMT2C, a hereditary motor and sensory neuropathy with diaphragm and vocal cord paresis (Dyck et al., 1994). Alterations in TRPV4 can have toxic consequences in neuronal cells due to changes in calcium concentrations (Deng et al., 2010). TRPV4 can induce autophagy through the AKT-pathway and potentially via regulation of calcium levels and osmotic pressure (Zhan et al., 2015). Furthermore, inhibition of TRPV4 by siRNA leads to inhibition of autophagy (Zhan et al., 2015). Therefore, dysregulation of autophagy is a potential pathomechanism in TRPV4 associated neuropathies, as a direct effect of mutant TRPV4 or as one of the neurotoxic consequences due to the disruption in calcium concentrations by TRPV4 mutants.

#### NDRG1 (N-myc Downstream Regulated 1)

N-myc downstream regulated 1 is mainly involved in cyto-protective stress response through regulation of p53 protein (Chen et al., 2010). In addition, it plays a role in immunity, development, differentiation and an important role in cancer (Fang et al., 2014). Mutations in *NDRG1* cause CMT4D neuropathy characterized with severe reduction in NCVs, skeletal and foot deformities and sensory loss (Kalaydjieva et al., 2000). NDRG1 inhibits basal and ER Stress induced autophagy via suppression of protein kinase-like endoplasmic reticulum kinase (PERK)/eIF2α axis (Sahni et al., 2014). Upregulation of NDRG1 has also been shown to initiate BNIP3 and Beclin mediated autophagy (Han et al., 2014). Autophagy regulation seems to be a part of the cyto-protective function of NDRG1. It may therefore play a role in fine tuning autophagy levels by acting on different autophagy inducing or inhibiting pathways. Though a link between CMT4D-causing *NDRG1* mutations and autophagy is yet to be established, mutations in *NDRG1* might influence autophagy regulation by NDRG1.

#### DNAJB2 [DnaJ Heat Shock Protein Family (Hsp40) Member B2, HSJ1]

HSJ1, a co-chaperone protein, is involved in binding ubiquitylated chaperone clients and their loading onto the Hsc70 chaperone, thus preventing aggregation and aiding proteasome sorting (Westhoff et al., 2005). HSJ1 consists of two isoforms with distinct intracellular localization. HSJ1a is cytoplasmic and nuclear, while HSJ1b localizes to the ER (Westhoff et al., 2005). In addition to its function in the proteasome system, HSJ1 has been shown to be involved in the recruitment of the autophagy marker protein LC3 to damaged mitochondria aiding in mitochondrial autophagy (mitophagy) (Rose et al., 2011) Recessive mutations in *HSJ1* affecting both isoforms can cause CMT2T neuropathy or purely motor AR-dHMN (Gess et al., 2014). HSJ1a, the cytoplasmic isoform has been shown to enhance autophagy and decrease aggregation (Novoselov et al., 2013; Sanchez et al., 2016). Furthermore, deletion mutations in HSJ1b isoform, have been shown to lead to neurodegeneration and irregular increase in the autophagy marker LC3 due to increase in HSJ1a expression (Sanchez et al., 2016). These studies present HSJ1 as a moderator of autophagy and proteasome activity and show that mutations in *HSJ1* could disrupt this balance leading to impaired clearance of ubiquitylated proteins and autophagy.

#### NEFL (Neurofilament Light)

Neurofilament light is part of the axoskeleton (neurofilaments) of large myelinated axons of the central and peripheral nerves. The main role of neurofilaments is maintaining fiber caliber and subsequently the conduction velocities of myelinated axons (Perrot et al., 2008). Mutations in *NEFL* can cause CMT2E/1F neuropathy with distal weakness and wasting of the lower limbs, and occasional cerebellar dysfunction, tremor, and hearing loss (Mersiyanova et al., 2000; Pareyson et al., 2014). CMT causing NEFL mutations have been shown to cause aggregate formation in cell culture and *in vitro* models. Activation of PKA, alleviates the aggregation phenotype (Sasaki et al., 2006). PKA is a known regulator of autophagy mostly exerting an inhibitory effect through phosphorylation of ATG13 an essential protein in the Ulk1 autophagy-initiation complex (Stephan et al., 2009), and of LC3 (Cherra et al., 2010). Therefore, the aggregation phenotype of NEFL might be a by-product of abnormality in the autophagy pathway which is restored by the activation of PKA. Furthermore, NEFL has been shown to interact with the PI3-phosphatase MTMR2 (Previtali et al., 2003). This interaction might be essential for the potential role of MTMR2 in autophagy through its regulation of endosomal vesicle trafficking or through its interaction with MTMR13, a direct regulator of autophagosome–lysosome fusion (see IPN Genes and Lysosomal Fusion and Degradation).

### IPN Genes and Autophagosome Formation and Expansion

#### TrkA (NTRK1) (Neurotrophic Receptor Tyrosine Kinase 1)

Tyrosine kinase A, a nerve growth factor (NGF) receptor, is essential for neuronal survival and regulation of neuronal death (Miller and Kaplan, 2001). TrkA has been associated with regulating the interplay between autophagy and apoptosis mainly in cancer cells (Hansen et al., 2007; Dadakhujaev et al., 2009). Overexpression of TrkA leads to an increase in the amount of ATG5-ATG12 conjugates and ultimately to an increase in autophagy marker LC3II (Dadakhujaev et al., 2009). Mutations in *TrkA* cause HSAN-IV neuropathy with congenital insensitivity to pain (CIPA) marked by absence of reaction to noxious stimuli and self-mutilating behavior (Indo et al., 1996). Recently, mutant TrkA has been shown to cause accumulation of autophagosomes. This accumulation proved to be a result of aberrant activation of autophagy and over saturation of the lysosomes rather than a deficit in autophagosome–lysosome fusion. Neurons expressing TrkA mutant show increased cell toxicity and dystrophic neurites as a result of autophagosomes accumulation by the abnormal autophagy activation (Franco et al., 2016).

#### DNM2 (Dynamin2)

Dynamin2 is one of the three isoforms of dynamin. DNM2 is a mechano-GTPase involved in endocytosis, Golgi function and vesicle trafficking (González-Jamett et al., 2013). DNM2 has been shown to play essential roles in autophagy. One of its main roles has been the autophagic lysosomal reformation, supplying nascent protolysosomes and hence maintaining the autophagic flux (Schulze and McNiven, 2014; Klionsky et al., 2016). Recently, DNM2 has been shown to interact with another protein Bif-1 to mediate the generation of Atg9-containing vesicles delivering Atg9 to autophagosome formation sites and promoting the formation of autophagosomes (Takahashi et al., 2016). Dominant mutations in *DNM2* have been linked with the intermediate form of CMT (DI-CMTB) with NCVs ranging from normal to 25 m/s (Züchner et al., 2005). A mutant DNM2 mouse model shows signs of neuropathy and a decreased autophagic flux indicated by lower levels of LC3II expression and P62-positive bodies [a selective autophagy marker also known as sequestosome1 (SQSTM1)] (Durieux et al., 2012). These studies suggest that mutations in *DNM2* leading to neuropathy affect its function in the formation of autophagosomes and present autophagy impairment as a pathomechanism of DNM2-linked neuropathy.

#### KIF1A (Kinesin Family Member 1A)

Kinesin family member 1A is a kinesin motor protein essential for the transport of vesicles in neuronal axons in anterograde fashion (Lo et al., 2011). Mutations in *KIF1A* can cause hereditary sensory and autonomic neuropathy type 2 (HSAN-II) (Rivire et al., 2011). HSAN-II patients suffer from frequent occurrence of unrecognized injuries and fractures of hands and feet due to loss in all peripheral sensations including pain and temperature. Autonomic symptoms include severe feeding problems in infants and common gastroesophageal reflux. Recently, it was shown that KIF1A controls the localization of ATG-9 and regulates the spatial distribution of autophagosomes in developing neurons of *Caenorhabditis elegans* (Stavoe et al., 2016). Maintaining ATG9 trafficking is essential for proper autophagosome formation (Lamb et al., 2016). The effects on autophagy in mutant KIF1A patients or cell/mouse models remains to be studied, but it is not inconceivable that impairment of autophagy due to disruption in ATG9 trafficking in KIF1A mutants is one of the pathomechanisms of neuropathy due to *KIF1A* mutations.

#### LITAF (Lipopolysaccharide-Induced TNF Factor)

Lipopolysaccharide-induced TNF factor, has been mainly linked with inflammatory functions, namely the secretion of cytokines such as TNF upon lipopolysaccharide stimulation (Tang et al., 2006). It has also been implicated in tumor suppression pathways and p53-induced apoptotic pathway (Zhou et al., 2011; Liu et al., 2012). LITAF seems to have different functions in different cell types. A role for LITAF in the positive regulation of autophagy has been shown in B cells. LITAF regulates LC3 expression and colocalizes with autophagosomes in B cells. Furthermore, overexpression of LITAF stimulates autophagy while silencing LITAF decreases the autophagic flux in these cells (Bertolo et al., 2013). Mutations in *LITAF* cause the dominant demyelinating CMT1C neuropathy (Street et al., 2003). In HEK293 cell models expressing CMT1C associated LITAF mutant protein, the mutant proteins cause mislocalisation of LITAF from early endosomes to the cytosol, destabilize LITAF and cause it to be aggregation prone. The aggregating mutant LITAF is then degraded by the proteasome and by autophagy (Lee et al., 2011). In neuroblastoma cell lines expressing WT and mutant forms of LITAF, CMT-causing LITAF mutants are mislocalised to the mitochondria, while the WT form traffics through the secretory pathway to the late endosome/lysosome (Lacerda et al., 2014). Late endosomes and lysosomes are essential for supplying the maturing autophagosome and for degrading the lysosomal content (Murrow et al., 2015). The trafficking function of LITAF might be related to its effect on autophagic flux, mislocalisation of mutant LITAF can therefore have a negative effect on the integrity of the endo/lysosomes and consequently on the autophagic process.

#### SH3TC2 (SH3 Domain and Tetratricopeptide Repeats 2)

*SH3TC2* encodes the Src homology 3 domain and tetratricopeptide repeats 2 protein. Little is known about the function of SH3TC2. However, the several motifs it contains suggest protein-protein interaction functions. Recessive mutations in *SH3TC2* cause a form of demyelinating neuropathy, CMT4C, characterized with severe scoliosis (Senderek et al., 2003b). Wild-type SH3TC2 has been shown to localize to recycling endosomes in rat Schwann cells, while the CMT causing mutant forms mistarget SH3TC2 away from the recycling endosomes (Roberts et al., 2010). SH3TC2 interacts with the small GTPase Rab11. The SH3TC2/Rab11 interaction is disrupted in mutant SH3TC2 in cultured cell lines (Roberts et al., 2010). Interestingly, KO of SH3TC2 in a transgenic mouse model decreases the expression of Rab11 in nerves of these mice (Stendel et al., 2010). Rab11 has been described as a positive regulator of autophagy. ULK1 and ATG9 localize in part to Rab11 positive recycling endosomes. Rab11 dependent vesicular transport from the recycling endosomes contributes to the forming autophagosomes and regulates starvation induced autophagy (Longatti et al., 2012). So by disrupting Rab11 interaction and expression levels and mistargeting SH3TC2 away from the recycling endosomes, *SH3TC2* CMT-associated mutations could disrupt the contribution of recycling endosomes to autophagosome formation.

#### TECPR2 (Tectonin Beta-Propeller Repeat Containing 2)

Tectonin beta-propeller repeat containing 2 was initially identified as human ATG8-interacting protein (Behrends et al., 2010). TECPR2 possesses an LC3 interacting motif (LIR) through which it binds to LC3B and LC3C family proteins. TECPR2 LIR-dependent binding leads to its association with cellular trafficking components such as HOPS and SEC24D. This interaction ultimately maintains a functional ERES and efficient ER export. TECPR2 depletion causes a decrease in ERES number and a delayed ER export. Maintaining a functional ERES provides a scaffold for autophagosome formation (Stadel et al., 2015). Mutations in *TECPR2* leading to the translation of a truncated and unstable version of TECPR2 cause a form of HSP (Oz-Levi et al., 2012) and hereditary sensory and autonomic neuropathy (HSAN-III) (Heimer et al., 2016). The latter presents with intellectual disability and, spasticity, and chronic respiratory disease. Skin fibroblasts from TECPR2-related HSP patients show a decreased number of LC3 and P62 proteins marking a decreased autophagic flux (Oz-Levi et al., 2012). These fibroblasts also show delayed ER export (Stadel et al., 2015), an indication that the function of TECPR2 in maintaining ER export and the formation of early autophagosome intermediates is disrupted by HSP-causing mutations, presenting autophagy disruption as a likely pathomechanism in HSP neuropathy.

### IPN Genes and Autophagosome Transport

#### DCTN1 (Dynactin Subunit 1)

Dynactin subunit 1 is the largest subunit of the dynactin complex. The dynactin complex has been associated with a large range of cellular functions including mitosis, ER-Golgi transport, lysosomes and endosomes movement as well as interacting with dynein for cargo transport (Schroer, 2004). Mutations in *DCTN1* disrupt axonal transport and lead to hereditary motor neuropathy (dHMN-VIIb) with breathing difficulty due to vocal fold paralysis and progressive facial weakness (Puls et al., 2003). DCTN1 knock-down shows motor neuron degeneration associated with autophagosomes accumulation due to impaired transport of autophagosomes along the axons (Ikenaka et al., 2013). Similarly, in a mouse model of mutant DCTN1, abnormal accumulation of autophagosomes has been shown in motor neurons and linked to the motor neuropathy phenotype (Wiesner et al., 2015). This presents autophagy impairment as a pathomechanism leading to motor neuropathy for *DCTN1* mutations.

#### DST (Dystonin)

The dystonin gene encodes several dystonin protein isoforms which are cytoskeletal cross-linking proteins that can interact with different organelles, microtubules, and protein complexes (Ferrier et al., 2013). Mutations in *DST* cause hereditary sensory and autonomic neuropathy (HSAN-VI) also known as familial dystonomia and presenting with alacrima, depressed deep tendon reflexes, and lingual fungiform papillae (Edvardson et al., 2012). In a mouse model of dystonia, mutant DST disrupts the autophagic process as evident by the accumulation of LC3 and P62 proteins. This impairment is attributed to the failure of autophagosome–lysosome fusion suggesting that dystonin is required to transport the autophagosomes to the lysosomes for maturation into autolysosomes (Ferrier et al., 2015). Interestingly, expression of the non-mutated form of dystonin restores the autophagy deficits revealing that the autophagy deficit is a direct effect of the mutated dystonin (Ferrier et al., 2015).

### IPN Genes and Lysosomal Fusion and Degradation

#### RAB7 (Member RAS Oncogene Family)

Ras-related protein Rab-21 is a small GTPase functioning in vesicular trafficking, more specifically in the transport from early to late endosomes and from late endosomes to lysosomes (Stenmark, 2009). RAB7 is recruited to autophagosomes and is required for the fusion of autophagosomes and lysosomes (Jäger et al., 2004). Mutations in *RAB7* cause CMT2B neuropathy characterized by distal muscle wasting and weakness and frequent foot ulcers and infections (Verhoeven et al., 2003). Dominant negative mutations retard the recruitment of RAB7 to autophagosomes, and prevent the progression of autophagy by impairing autolysosome formation (Gutierrez et al., 2004).

#### CCT5 (Chaperonin Containing TCP-1 Subunit 5)

A subunit of the TCP-1 containing chaperonin complex (CCT), an ATP-dependent chaperone responsible for folding unfolded proteins including actin and tubulin. Recessive mutations in *CCT5* cause a mutilating hereditary sensory and autonomic neuropathy with spastic paraplegia (HSNSP) (Bouhouche et al., 2006). Studies have shown that CCT can prevent mutant huntingtin (htt) aggregation *in vitro* (Darrow et al., 2015). Recently, it was shown that the function of CCT in preventing aggregation of mutant proteins is mediated by autophagy (Pavel et al., 2016). CCT is required for lysosomal biogenesis and functioning and for autophagosome–lysosome fusion, possibly via interaction with cytoskeleton proteins. Disruption of CCT integrity by knock-down or by mutations in *CCT5* disrupts autolysosome formation and cargo degradation (Pavel et al., 2016).

#### FIG4 (FIG4 Phosphoinositide 5-Phosphatase)

FIG4 phosphoinositide 5-phosphatase is a phospholipid phosphatase responsible for the generation and turnover of the PtdIns(3,5)P2 phosphoinositide (Di Paolo and De Camilli, 2006). Mutations in *FIG4* cause Yunis–Varon syndrome, familial epilepsy with polymicrogyria, and the severely demyelinating Charcot-Marie-Tooth type 4J neuropathy (CMT4J) (Chow et al., 2007; Katona et al., 2011; Vaccari et al., 2015). In a FIG4 deficient neuropathic mouse model, sensory and motor neurons as well as Schwann cells seem to be affected (Katona et al., 2011; Vaccari et al., 2015). Neurons show decrease of PtdIns(3,5)P2 levels, a sign of impaired FIG4 enzymatic activity, and enlargement of late endosomes and lysosomes. Enlargement of late endosomes and lysosomes can hinder the fusion capacity of autophagosomes with lysosomes. Indeed these mice show accumulation of autophagic markers LC3II and P62 in their sciatic nerve. This accumulation is independent of an increase in the autophagic flux (Ferguson et al., 2010; Vaccari et al., 2015). Thus abolishing the enzymatic activity of FIG4 by missense mutations or haploinsufficiency seems to affect the progression of autophagic degradation by preventing the fusion between autophagosomes and the then enlarged late endosomes/lysosomes (Vaccari et al., 2015).

#### MTMRs (Myotubularin-Related Proteins)

Myotubularin-related proteins are PI3-phosphatases consisting of catalytically active or inactive members. Mutations in *MTMR2* and *MTMR13* (also called SET-binding factor 2) cause the demyelinating CMT4B1 and CMT4B2 neuropathies respectively with early-onset glaucoma (Azzedine et al., 2003). MTMR2 is a catalytically active phosphatase which dephosphorylates phosphatidylinositol 3-phosphate (PI(3)P) and bisphosphate PI(3,5)P2. MTMR13 on the other hand is an inactive phosphatase but it has been shown to associate with MTMR2 (Robinson and Dixon, 2005). MTMR2 has been linked with regulation of the late endocytic pathway and vesicular transport through its putative substrate PI(3,5)P2 (Bolino et al., 2004; Lenk and Meisler, 2014). MTMR13 on the other hand acts as a RAB21 GEF required for fusion of autophagosome with the lysosome. MTMR13 GEF activity is induced upon starvation and it promotes the trafficking of VAMP8 to the lysosome where it is needed to mediate fusion with the autophagosome (Jean et al., 2015). Autophagy has not yet been studied in the context of CMT4B causing mutations in *MTMRs* but the roles of both MTMR2 and MTMR13 in autophagy and their functional association with each other presents autophagy impairment as a possible common pathomechanism in mutant MTMRs leading to neuropathy.

#### VCP (Valosin Containing Protein)

Valosin containing protein is an AAA+ ATPase associated with diverse cellular activities including the ATP-dependent remodeling of proteins to unfold them or extract them from cell structures or binding partners (Erzberger and Berger, 2006). Mutations in *VCP* cause a multisystem degenerative disease consisting of inclusion body myopathy, Paget’s diseases and frontotemporal dementia (IBMPFD) (Kimonis et al., 2000), familial ALS (Shaw, 2010) and can also lead to CMT2Y neuropathy with distal muscle weakness and atrophy and length-dependent sensory loss (Gonzalez et al., 2014). VCP also plays a role in lysosomal homeostasis and the lysosomal damage response. Lysosomal damage induced by lysomotropic reagents triggers a concomitant recruitment of VCP to lysosomes. VCP then cooperates with a set of cofactors to drive the degradation of ruptured lysosomes (Papadopoulos et al., 2016). Damaged lysosomes accumulate in cells expressing mutant VCP showing that its role in clearing out damaged lysosomes is compromised by neuropathy- causing mutations. VCP mutant knock-in mouse model shows accumulation of LC3II positive structures in myoblasts (Nalbandian et al., 2013). Furthermore, knock-down of VCP leads to accumulation of autophagosomes due their failure to mature into autolysosomes (Ju et al., 2009). A high percentage of the accumulated autophagic vesicles contain ubiquitin positive structures. VCP has been shown to be critical for the maturation of ubiquitin-containing autophagosomes into autolysosomes under basal conditions and under proteasome inhibition. This function of VCP in maturation of autophagosomes seems to be impaired by neuropathy-causing mutations (Lee et al., 2010).

#### HSPB8 (Heat Shock Protein B8)

Heat shock protein B8 belongs to the family of small heat shock proteins, ATP-independent chaperones that aid the folding of misfolded proteins by ATP-dependent chaperones (Holmgren, 2012). In addition to its function as a molecular chaperone, HSPB8 is involved in several other stress signaling functions such as removal of aggregates through CASA (Carra et al., 2008). CASA involves the recognition of substrate proteins by a complex of chaperones and co-chaperones including HSPB8 and P62 and its subsequent loading onto autophagosomes. Mutations in *HSPB8* cause CMT2L and dHMN-IIa neuropathies typically presenting with paresis of the extensor muscles of the big toe and then the feet (Irobi et al., 2004; Tang et al., 2005). Expression of mutant HSPB8 in cultured cells induces aggregate formation (Irobi et al., 2004). Transcriptional induction of WT HSPB8 in mouse models of motor neuron disease aids in the clearance of misfolded proteins and aggregates (Crippa et al., 2016). Interestingly, cells from neuropathic patients carrying HSPB8 mutations also show deficits in lysosomal delivery of autophagosomes (Kwok et al., 2011). Taken together, these studies indicate that intact HSPB8 is necessary for aggregate clearance, possibly through CASA. Disruption of this role by mutations in *HSPB8* contribute to the neuronal pathogenicity.

### IPN Genes Involved in Selective Autophagy: Mitophagy, Reticulophagy (ER-Phagy)

#### FAM134B (Family with Sequence Similarity 134 Member B)

Family with sequence similarity 134 member B is a transmembrane protein localized to the *cis*-golgi and predominantly expressed in the sensory and autonomic ganglia. Mutations in *FAM134B* cause hereditary sensory and autonomic neuropathy HSAN-IIB leading to impaired nociception, autonomic dysfunction, and severe mutilations (Kurth et al., 2009). FAM134B interacts with LC3/GABARAP through an LC3 interacting motif at its C-terminal domain. This interaction is essential for selectively directing part of the ER to autophagosomes (reticulophagy). Mutations causing sensory neuropathy disrupt the LC3 interacting motif and consequently the FAM134B-LC3 binding. FAM134B KO mice develop sensory neuropathy and degeneration of sensory neuronal axons. Deficit in reticulophagy in these mice but not general autophagy disrupts ER homeostasis leading to ER expansion and inhibition of ER turnover that ultimately leads to degeneration of sensory neurons (Khaminets et al., 2015).

#### MFN2 (Mitofusin 2)

Mitofusin 2 was identified as a transmembrane mitochondrial GTPase required for mitochondrial fusion (Santel and Fuller, 2001). Mutations in *MFN2* cause CMT2A neuropathy (Züchner et al., 2004) and are one of the most frequent CMT2 causing mutations (OMIM 609260, CMT2A ≈ 35% of CMT2). CMT2A presents as a severe predominantly motor neuropathy or motor accompanied with profound proprioception loss. In addition to its role in mitochondrial fusion, MFN2 plays multiple roles including regulation of cell survival, cell proliferation, ER stress and autophagy (reviewed in Schrepfer and Scorrano, 2016). *MFN2* deletion has been shown to impair starvation-induced autophagy by disrupting the MAM (mitochondria associated membranes), a described site for autophagosome formation (Hailey et al., 2010; Hamasaki et al., 2013). *MFN2* deletion in HeLa cells has also been shown to affect cell proliferation due to autophagy impairment (Ding et al., 2015). Motor neurons derived from iPSCs obtained from CMT2A patients show increase in mitophagy and mitochondrial depletion and increased expression of *PINK1, PARK2* and BNIP3, known triggers for autophagic degradation of mitochondria (Rizzo et al., 2016). In a MFN2 KO mouse model, sarcopenia is seen in correlation with impaired autophagy in the muscle, accumulation of damaged mitochondria, and activation of an adaptive mitophagy pathway (Sebastián et al., 2016). Taken together, MFN2 seems to play an essential role in autophagosome formation, possibly through maintenance of mitochondrial membranes and MAMs. This role is impaired in MFN2 deficiency and in MFN2 causing CMT2A mutations, leading to autophagy impairment and disruption of balance between mitophagy and general autophagy.

#### GDAP1 (Ganglioside Induced Differentiation Associated Protein 1)

Ganglioside induced differentiation associated protein 1 is an integral protein of the outer mitochondrial membrane formed of 2 glutathione-*S*-transferase domains (Huber et al., 2016). Dominant and recessive mutations in *GDAP1* cause demyelinating (CMT4A), or axonal (AR-CMT2 or CMT2K) neuropathies (Baxter et al., 2002; Senderek et al., 2003a; Sivera et al., 2010). GDAP1 is required for the regulation of the mitochondrial network and mitochondrial integrity (Niemann et al., 2005). In a KO mouse model of GDAP1 that recapitulates the neuropathic phenotype of CMT4A, disruption of the mitochondrial network and mitochondrial calcium homeostasis is evident in neurons and associates with accumulation of autophagic vesicles (Barneo-Muñoz et al., 2015). Mitochondrial defects can lead to abnormal increase in targeting mitochondria to autophagic degradation (mitophagy). In addition, both mitochondrial interconnectivity and calcium homeostasis are essential for the proper functioning of autophagy (Gomez-Suaga et al., 2017). It remains unclear whether the increased accumulation of autophagosomes in neurons of CMT4A mouse models is a by-product of increased mitophagy or defects in the general autophagic flux. Nonetheless, autophagic dysregulation does seem to be a feature of the pathology caused by CMT-causing *GDAP1* mutations and might explain the variety of clinical phenotypes presented by these mutations ranging from myelinating to axonal CMT and from dominant to recessive.

## Autophagy Modulation as a Therapeutic Target in Hereditary Neuropathies

So far, finding a treatment for hereditary neuropathies remains a challenge. An important step toward a successful therapy, from both an economic and a pharmaceutical perspective, is finding a common pathomechanism that can be targeted in different neuropathic genotypes. By bridging different neuropathic phenotypes and different causal genes, autophagy impairment emerges as a good candidate for modulation by therapeutic measures. Autophagy modulation as a therapy approach has been used in several more common neurodegenerative disease models. Inducing autophagy has been shown to reduce the severity of the Huntington’s disease phenotype and improve clearance of Htt aggregates in mouse and neuronal cell models (Rose et al., 2010). In Alzheimer’s disease, accumulation of autophagosomes contributes to the pathology (Ohta et al., 2010). Inhibition of autophagy has been reported as a mode of action in drugs that decrease amyloid-β accumulation in Alzheimer’s patients (Lipinski et al., 2010). Accumulation of autophagosomes has also been attributed to defects in beclin-1 expression, hence autophagy enhancing treatments such as resveratrol and lithium can improve the pathology of Alzheimer’s disease (Vingtdeux et al., 2010; Rahvar et al., 2011). In addition, expression of beclin-1 in neuronal cells of transgenic mice improves their Parkinson’s disease phenotype by enhancing lysosomal activation (Spencer et al., 2009). Similarly, the mTOR-independent autophagy inducer trehalose improves the clearance of α-synuclein, huntingtin (Sarkar et al., 2007) and SOD1 aggregates (Castillo et al., 2013a). Furthermore, autophagy modulating compounds are already in use to treat different neurodegenerative diseases and cancers, and are currently at different phases of clinical trials (reviewed in Towers and Thorburn, 2016). Despite its promise, autophagy modulation as a treatment for hereditary neuropathies still presents many challenges. The model of treating mutants that increase autophagic activation with inhibitors of autophagy and those that inhibit autophagy with activators is oversimplified. Many autophagy modulating drugs can have off-target effects. For example, mTORC1 inhibitors can affect cell metabolism and lipid synthesis in an autophagy-independent manner (Li et al., 2014). Therefore, targeting the specific autophagy step affected by a hereditary neuropathy causing-mutation is a better and more elegant approach to enhance the autophagic status without hindering other vital cellular processes. Certain step-specific approaches already exist and include direct stimulation of autophagy by targeting beclin1 with the alkaloid isorhynchophylline (Lu et al., 2012). Targeting the lysosomal fusion and degradation is also possible using pharmacological activators of lysosomal enzymes such ambroxol (McNeill et al., 2014) or acidic nanoparticles that increase the acidity of the lysosomes (Baltazar et al., 2012). Non-pharmacological enhancement of autophagy such as caloric restriction or exercise presents another way of overcoming the pharmacological modulation of other cellular pathways. Physical exercise has already been described as a rehabilitating measure in CMT patients (Roberts-Clarke et al., 2016; Vita et al., 2016). Whether improvement of autophagy is a mediator of the positive impact of exercise remains to be investigated, but exercise has been shown to enhance autophagy in the brain (He et al., 2012) and skeletal muscles (Ferraro et al., 2014). Another caveat of autophagy directed therapy is the difficulty of tracing autophagy *in vivo*. In other words, there is a need for biomarkers that can be used to make sure that the intended therapy is improving the autophagic status in patients in order to alleviate their symptoms. Such markers are as of yet unavailable, but recent advances using mouse models allows following up a treatment’s effect on autophagy in mouse tissues (Castillo et al., 2013b). This strategy, combined to behavioral and electrophysiological testing in a neuropathic mouse model can permit the direct correlation between a drug’s effect on autophagy and its ultimate modulation of the neuropathic status laying out a strong case for such a drug to enter clinical trials.

## Conclusion

The genetic heterogeneity of IPNs and the fact that they are rare disorders, highlights the need for finding common cellular and molecular pathomechanisms among the different disease-linked genes. This review complements recent work in attempting to find shared molecular mechanisms underlying IPNs (Bucci et al., 2012; Prior et al., 2017). The relevance of autophagy as a target pathway stems from the fact that it has been implicated in other disease conditions, mainly neurodegeneration, where autophagy targeting drugs have already entered clinical trials. In addition, autophagic impairment presents a unique overlap among neuropathies affecting both sensory and motor nerves on one hand, and the myelin sheath and the neuronal axon on the other. While more research is required to get a clearer view of the involvement of autophagy in neuropathic mechanisms, current evidence points out to the emergence of autophagy as a frequently affected pathway in IPN conditions.

## Author Contributions

MH wrote the manuscript and designed table and figures. VT supervised and recommended the content of the manuscript, corrected, and submitted.

## Conflict of Interest Statement

The authors declare that the research was conducted in the absence of any commercial or financial relationships that could be construed as a potential conflict of interest.

## Abbreviations

ALS: amyotrophic lateral sclerosis
ATG: autophagy-related gene
BCL2: B-cell lymphoma2 (BCL2) apoptosis regulator
BECN1: beclin 1
BNIP3: BCL2 interacting protein 3
BSCL2: seipin lipid droplet biogenesis associated
CASA: chaperone-assisted selective autophagy
CCT5: chaperonin containing TCP1 subunit 5
CMA: chaperone mediated autophagy
CMAP: compound muscle action potential
CMT: Charcot-Marie-Tooth
DCTN1: dynactin subunit 1
dHMN: distal hereditary motor neuropathy
DNAJB2: DnaJ heat shock protein family (Hsp40) member B2
DNM2: dynamin2
DST: dystonin
eIF2α: eukaryotic initiation factor 2-alpha
ER: endoplasmic reticulum
ERES: ER exit sites
FAM134B: family with sequence similarity 134 member B
FIG4: FIG4 phosphoinositide 5-phosphatase
GABARAP: GABA type A receptor-associated protein
GDAP1: ganglioside induced differentiation associated protein 1
GEF: guanine nucleotide exchange factor
HOPS: homotypic fusion and protein sorting
HSAN: hereditary sensory and autonomic neuropathy
HSP: hereditary spastic paraplegia
HSPB8: heat shock protein B8
IPNs: inherited peripheral neuropathies
iPSCs: induced pluripotential stem cells
KIF1A: kinesin family member 1A
KO: knock-out
LC3: microtubule associated protein 1 light chain 3 (MAP1LC3)
LITAF: lipopolysaccharide induced TNF factor
LRSAM1: leucine rich repeat and sterile alpha motif containing 1
MFN2: mitofusin 2
MTMR2/13: myotubularin-related protein 2/13
MTORC1: mechanistic target of rapamycin 1
NCVs: nerve conduction velocities
NDRG1: N-myc downstream regulated 1
NEFL: neurofilament light
P62/SQSTM1: sequestosome 1
PARK2: parkin 2
PERK: protein kinase R (PKR)-like endoplasmic reticulum kinase
PHF23: PHD finger protein 23
PIK3C3/VPS34: phosphatidylinositol 3-kinase catalytic subunit type 3
PIK3R4/VPS15: phosphoinositide-3-kinase regulatory subunit 4
PINK1 PTEN: induced putative kinase 1
PKA: protein kinase A
PMP22: peripheral myelin protein 22
PtdIns: phosphatidyl inositol
PTPIP51: protein tyrosine phosphatase interacting protein 51
Rab11: Ras-related protein Rab-11A
RAB21: Ras-related protein Rab-7
RAB7: Ras-related protein Rab-21
RB1CC1: RB1 inducible coiled-coil 1
SEC24D: SEC24 homolog D, COPII coat complex component
SH3TC2: SH3 domain and tetratricopeptide repeats 2
SNARE: SNAP (soluble NSF attachment protein) receptor
SOD1: superoxide dismutase 1
TECPR2: tectonin beta-propeller repeat containing 2
TrkA: tyrosine kinase A
TRPV4: transient receptor potential cation channel subfamily V member 4
ULK1/2: unc-51 like autophagy activating kinase 1/2
UVRAG: UV radiation resistance associated
VAPB: VAMP associated protein B
VCP: valosin containing protein
WNK1: WNK lysine deficient protein kinase 1
WT: wild-type

## References

[B1] Amit I. Yakir L. Katz M. Zwang Y. Marmor M. D. Citri A. (2004). Tal, a Tsg101-specific E3 ubiquitin ligase, regulates receptor endocytosis and retrovirus budding. *Genes Dev.* 18 1737–1752. 10.1101/gad.294904 15256501 PMC478194

[B2] Axe E. L. Walker S. A. Manifava M. Chandra P. Roderick H. L. Habermann A. (2008). Autophagosome formation from membrane compartments enriched in phosphatidylinositol 3-phosphate and dynamically connected to the endoplasmic reticulum. *J. Cell Biol.* 182 685–701. 10.1083/jcb.200803137 18725538 PMC2518708

[B3] Azzedine H. Bolino A. Taïeb T. Birouk N. Di Duca M. Bouhouche A. (2003). Mutations in MTMR13, a new pseudophosphatase homologue of MTMR2 and Sbf1, in two families with an autosomal recessive demyelinating form of Charcot-Marie-Tooth disease associated with early-onset glaucoma. *Am. J. Hum. Genet.* 72 1141–1153. 10.1086/375034 12687498 PMC1180267

[B4] Baets J. Jonghe P. De Timmerman V. (2014). Recent advances in Charcot-Marie-Tooth disease. *Curr. Opin. Neurol.* 27 532–540. 10.1097/WCO.0000000000000131 25110935

[B5] Baltazar G. C. Guha S. Lu W. Lim J. Boesze-Battaglia K. Laties A. M. (2012). Acidic nanoparticles are trafficked to lysosomes and restore an acidic lysosomal pH and degradative function to compromised ARPE-19 cells. *PLoS ONE* 7:e49635 10.1371/journal.pone.0049635 PMC3525582 23272048

[B6] Barneo-Muñoz M. Juárez P. Civera-Tregón A. Yndriago L. Pla-Martín D. Zenker J. (2015). Lack of GDAP1 induces neuronal calcium and mitochondrial defects in a knockout mouse model of Charcot-Marie-Tooth neuropathy. *PLOS Genet.* 11:e1005115 10.1371/journal.pgen.1005115 PMC4393229 25860513

[B7] Baxter R. V. Ben Othmane K. Rochelle J. M. Stajich J. E. Hulette C. Dew-Knight S. (2002). Ganglioside-induced differentiation-associated protein-1 is mutant in Charcot-Marie-Tooth disease type 4A/8q21. *Nat. Genet.* 30 21–22. 10.1038/ng796 11743579

[B8] Behrends C. Sowa M. E. Gygi S. P. Harper J. W. (2010). Network organization of the human autophagy system. *Nature* 466 68–76. 10.1038/nature09204 20562859 PMC2901998

[B9] Bertolo C. Roa S. Sagardoy A. Mena-Varas M. Robles E. F. Martinez-Ferrandis J. I. (2013). LITAF, a BCL6 target gene, regulates autophagy in mature B-cell lymphomas. *Br. J. Haematol.* 162 621–630. 10.1111/bjh.12440 23795761 PMC4111142

[B10] Bolino A. Bolis A. Previtali S. C. Dina G. Bussini S. Dati G. (2004). Disruption of Mtmr2 CMT4B1-like neuropathy with myelin outfolding and impaired spermatogenesis. *J. Cell Biol.* 167 711–721. 10.1083/jcb.200407010 15557122 PMC2172586

[B11] Bouhouche A. Benomar A. Bouslam N. Chkili T. Yahyaoui M. (2006). Mutation in the epsilon subunit of the cytosolic chaperonin-containing t-complex peptide-1 (Cct5) gene causes autosomal recessive mutilating sensory neuropathy with spastic paraplegia. *J. Med. Genet.* 43 441–443. 10.1136/jmg.2005.039230 16399879 PMC2564519

[B12] Bucci C. Bakke O. Progida C. (2012). Charcot-Marie-Tooth disease and intracellular traffic. *Prog. Neurobiol.* 99 191–225. 10.1016/j.pneurobio.2012.03.003 22465036 PMC3514635

[B13] Carra S. Seguin S. J. Lambert H. Landry J. (2008). HspB8 chaperone activity toward poly(Q)-containing proteins depends on its association with Bag3, a stimulator of macroautophagy. *J. Biol. Chem.* 283 1437–1444. 10.1074/jbc.M706304200 18006506

[B14] Cartwright B. R. Goodman J. M. (2012). Seipin: from human disease to molecular mechanism. *J. Lipid Res.* 53 1042–1055. 10.1194/jlr.R023754 22474068 PMC3351812

[B15] Castillo K. Nassif M. Valenzuela V. Rojas F. Matus S. Mercado G. (2013a). Trehalose delays the progression of amyotrophic lateral sclerosis by enhancing autophagy in motoneurons. *Autophagy* 9 1308–1320. 10.4161/auto.25188 23851366

[B16] Castillo K. Valenzuela V. Matus S. Nassif M. Oñate M. Fuentealba Y. (2013b). Measurement of autophagy flux in the nervous system in vivo. *Cell Death Dis.* 4 e917 10.1038/cddis.2013.421 PMC3847309 24232093

[B17] Chen B. Longtine M. S. Sadovsky Y. Nelson D. M. (2010). Hypoxia downregulates p53 but induces apoptosis and enhances expression of BAD in cultures of human syncytiotrophoblasts. *Am. J. Physiol. Cell Physiol.* 299 C968–C976. 10.1152/ajpcell.00154.2010 20810912 PMC2980304

[B18] Chen Y. Klionsky D. J. (2011). The regulation of autophagy - unanswered questions. *J. Cell Sci.* 124 161–170. 10.1242/jcs.064576 21187343 PMC3037094

[B19] Cherra S. J. Kulich S. M. Uechi G. Balasubramani M. Mountzouris J. Day B. W. (2010). Regulation of the autophagy protein LC3 by phosphorylation. *J. Cell Biol.* 190 533–539. 10.1083/jcb.201002108 20713600 PMC2928022

[B20] Chow C. Y. Zhang Y. Dowling J. J. Jin N. Adamska M. Shiga K. (2007). Mutation of FIG4 causes neurodegeneration in the pale tremor mouse and patients with CMT4J. *Nature* 448 68–72. 10.1038/nature05876 17572665 PMC2271033

[B21] Crippa V. D’Agostino V. G. Cristofani R. Rusmini P. Cicardi M. E. Messi E. (2016). Transcriptional induction of the heat shock protein B8 mediates the clearance of misfolded proteins responsible for motor neuron diseases. *Sci. Rep.* 6:22827 10.1038/srep22827 PMC4785366 26961006

[B22] Cuervo A. M. Palmer A. Rivett A. J. Knecht E. (1995). Degradation of proteasomes by lysosomes in rat liver. *Eur. J. Biochem.* 227 792–800. 10.1111/j.1432-1033.1995.tb20203.x 7867640

[B23] Dadakhujaev S. Eun J. J. Hae S. N. Hah Y. S. Chang J. K. Deok R. K. (2009). Interplay between autophagy and apoptosis in TrkA-induced cell death. *Autophagy* 5 103–105. 10.1016/j.yexcr.2008.08.013 19115484

[B24] Darrow M. C. Sergeeva O. A. Isas J. M. Galaz-Montoya J. G. King J. A. Langen R. (2015). Structural mechanisms of mutant huntingtin aggregation suppression by the synthetic chaperonin-like CCT5 complex explained by cryoelectron tomography. *J. Biol. Chem.* 290 17451–17461. 10.1074/jbc.M115.655373 25995452 PMC4498080

[B25] Deng H.-X. Klein C. J. Yan J. Shi Y. Wu Y. Fecto F. (2010). Scapuloperoneal spinal muscular atrophy and CMT2C are allelic disorders caused by alterations in TRPV4. *Nat. Genet.* 42 165–169. 10.1038/ng.509 20037587 PMC3786192

[B26] Di Paolo G. De Camilli P. (2006). Phosphoinositides in cell regulation and membrane dynamics. *Nature* 443 651–657. 10.1038/nature05185 17035995

[B27] Ding Y. Gao H. Zhao L. Wang X. Zheng M. (2015). Mitofusin 2-deficiency suppresses cell proliferation through disturbance of autophagy. *PLoS ONE* 10:e0121328 10.1371/journal.pone.0121328 PMC4363693 25781899

[B28] Durieux A. C. Vassilopoulos S. Lainé J. Fraysse B. Briñas L. Prudhon B. (2012). A centronuclear myopathy - dynamin 2 mutation impairs autophagy in mice. *Traffic* 13 869–879. 10.1111/j.1600-0854.2012.01348.x 22369075

[B29] Dyck P. J. Litchy W. J. Minnerath S. Bird T. D. Chance P. F. Schaid D. J. (1994). Hereditary motor and sensory neuropathy with diaphragm and vocal cord paresis. *Ann. Neurol.* 35 608–615. 10.1002/ana.410350515 8179305

[B30] Edvardson S. Cinnamon Y. Jalas C. Shaag A. Maayan C. Axelrod F. B. (2012). Hereditary sensory autonomic neuropathy caused by a mutation in dystonin. *Ann. Neurol.* 71 569–572. 10.1002/ana.23524 22522446

[B31] Erzberger J. P. Berger J. M. (2006). Evolutionary relationships and structural mechanisms of Aaa+ proteins. *Annu. Rev. Biophys. Biomol. Struct.* 35 93–114. 10.1146/annurev.biophys.35.040405.101933 16689629

[B32] Fan H. Chen S. Sun Y. Xu S. Wu L. (2015). Seipin mutation at glycosylation sites activates autophagy in transfected cells via abnormal large lipid droplets generation. *Acta Pharmacol. Sin.* 36 497–506. 10.1038/aps.2014.164 25832430 PMC4387305

[B33] Fang B. A. Kovačević Ž. Park K. C. Kalinowski D. S. Jansson P. J. Lane D. J. R. (2014). Molecular functions of the iron-regulated metastasis suppressor, NDRG1, and its potential as a molecular target for cancer therapy. *Biochim. Biophys. Acta* 1845 1–19. 10.1016/j.bbcan.2013.11.002 24269900

[B34] Ferguson C. J. Lenk G. M. Meisler M. H. (2010). PtdIns(3,5)P2 and autophagy in mouse models of neurodegeneration. *Autophagy* 6 170–171. 10.4161/auto.6.1.10626 20009544 PMC2859463

[B35] Ferraro E. Giammarioli A. M. Chiandotto S. Spoletini I. Rosano G. (2014). Exercise-induced skeletal muscle remodeling and metabolic adaptation: redox signaling and role of autophagy. *Antioxid. Redox Signal.* 21 154–176. 10.1089/ars.2013.5773 24450966 PMC4048572

[B36] Ferrier A. Boyer J. G. Kothary R. (2013). *Cellular and Molecular Biology of Neuronal Dystonin.* Amsterdam: Elsevier 10.1016/B978-0-12-405210-9.00003-5 23273860

[B37] Ferrier A. De Repentigny Y. Lynch-Godrei A. Gibeault S. Eid W. Kuo D. (2015). Disruption in the autophagic process underlies the sensory neuropathy in dystonia musculorum mice. *Autophagy* 11 1025–1036. 10.1080/15548627.2015.1052207 26043942 PMC4590603

[B38] Fortun J. Go J. C. Li J. Amici S. A. Dunn W. A. Notterpek L. (2006). Alterations in degradative pathways and protein aggregation in a neuropathy model based on PMP22 overexpression. *Neurobiol. Dis.* 22 153–164. 10.1016/j.nbd.2005.10.010 16326107

[B39] Frake R. A. Ricketts T. Menzies F. M. Rubinsztein D. C. (2015). Autophagy and neurodegeneration. *J. Clin. Invest.* 125 65–74. 10.1172/JCI73944 25654552 PMC4382230

[B40] Franco M. L. Melero C. Sarasola E. Acebo P. Luque A. Calatayud-Baselga I. (2016). Mutations in TrkA causing congenital insensitivity to pain with anhidrosis (CIPA) induce misfolding, aggregation, and mutation-dependent neurodegeneration by dysfunction of the autophagic flux. *J. Biol. Chem.* 291 21363–21374. 10.1074/jbc.M116.722587 27551041 PMC5076807

[B41] Gallolu Kankanamalage S. Lee A.-Y. Wichaidit C. Lorente-Rodriguez A. Shah A. M. Stippec S. (2016). Multistep regulation of autophagy by WNK1. *Proc. Natl. Acad. Sci. U.S.A.* 113 201617649 10.1073/pnas.1617649113 PMC5167150 27911840

[B42] Gess B. Auer-Grumbach M. Schirmacher A. Strom T. Zitzelsberger M. Rudnik-Schöneborn S. (2014). Ovid: HSJ1-related hereditary neuropathies: novel mutations and extended clinical spectrum. *Neurology* 83 1726–1732. 10.1212/WNL.0000000000000966 25274842

[B43] Gomez-Suaga P. Paillusson S. Stoica R. Noble W. Hanger D. P. Miller C. C. J. (2017). The ER-mitochondria tethering complex VAPB-PTPIP51 regulates autophagy. *Curr. Biol.* 27 371–385. 10.1016/j.cub.2016.12.038 28132811 PMC5300905

[B44] Gonzalez M. A. Feely S. M. Speziani F. Strickland A. V. Danzi M. Bacon C. (2014). A novel mutation in VCP causes Charcot-Marie-Tooth Type 2 disease. *Brain* 137 2897–2902. 10.1093/brain/awu224 25125609 PMC4208462

[B45] González-Jamett A. M. Momboisse F. Haro-Acuña V. Bevilacqua J. A. Caviedes P. Cárdenas A. M. (2013). Dynamin-2 function and dysfunction along the secretory pathway. *Front. Endocrinol.* 4:126 10.3389/fendo.2013.00126 PMC3776141 24065954

[B46] Guo J. Qiu W. Soh S. L. Y. Wei S. Radda G. K. Ong W.-Y. (2013). Motor neuron degeneration in a mouse model of seipinopathy. *Cell Death Dis.* 4 e535 10.1038/cddis.2013.64 PMC3613842 23470542

[B47] Gutierrez M. G. Munafó D. B. Berón W. Colombo M. I. (2004). Rab7 is required for the normal progression of the autophagic pathway in mammalian cells. *J. Cell Sci.* 117 2687–2697. 10.1242/jcs.01114 15138286

[B48] Hailey D. W. Rambold A. S. Satpute-Krishnan P. Mitra K. Sougrat R. Kim P. K. (2010). Mitochondria supply membranes for autophagosome biogenesis during starvation. *Cell* 141 656–667. 10.1016/j.cell.2010.04.009 20478256 PMC3059894

[B49] Hamasaki M. Furuta N. Matsuda A. Nezu A. Yamamoto A. Fujita N. (2013). Autophagosomes form at ER-mitochondria contact sites. *Nature* 495 389–393. 10.1038/nature11910 23455425

[B50] Han B. Li W. Sun Y. Zhou L. Xu Y. Zhao X. (2014). A prolyl-hydroxylase inhibitor, ethyl-34-Dihydroxybenzoate, induces cell autophagy and apoptosis in esophageal squamous cell carcinoma cells via up-regulation of BNIP3 and N-myc downstream-regulated gene-1. *PLoS ONE* 9:e107204 10.1371/journal.pone.0107204 PMC4169646 25232961

[B51] Hansen K. Wagner B. Hamel W. Schweizer M. Haag F. Westphal M. (2007). Autophagic cell death induced by TrkA receptor activation in human glioblastoma cells. *J. Neurochem.* 103 259–275. 10.1111/j.1471-4159.2007.04753.x 17635673

[B52] Hara T. Nakamura K. Matsui M. Yamamoto A. Nakahara Y. Suzuki-Migishima R. (2006). Suppression of basal autophagy in neural cells causes neurodegenerative disease in mice. *Nature* 441 885–889. 10.1038/nature04724 16625204

[B53] Harding A. E. Thomas P. K. (1980a). Hereditary distal spinal muscular atrophy: a report on 34 cases and a review of the literature. *J. Neurol. Sci.* 45 337–348. 7365507 10.1016/0022-510x(80)90177-x

[B54] Harding A. E. Thomas P. K. (1980b). Genetic aspects of hereditary motor and sensory neuropathy (types I and II). *J. Med. Genet.* 17 329–336. 10.1136/jmg.17.5.329 7218272 PMC1048594

[B55] He C. Sumpter R. Levine B. (2012). Exercise induces autophagy in peripheral tissues and in the brain. *Autophagy* 8 1548–1551. 10.4161/auto.21327 22892563 PMC3463459

[B56] Heimer G. Oz-Levi D. Eyal E. Edvardson S. Nissenkorn A. Ruzzo E. K. (2016). TECPR2 mutations cause a new subtype of familial dysautonomia like hereditary sensory autonomic neuropathy with intellectual disability. *Eur. J. Paediatr. Neurol.* 20 69–79. 10.1016/j.ejpn.2015.10.003 26542466

[B57] Holmgren A. (2012). *Molecular Biology of Small Heat Shock Proteins associated with Peripheral Neuropathies. eLS.*. Chichester: John Wiley Sons Ltd 1–10. 10.1002/9780470015902.a0024294

[B58] Hosokawa N. Hara T. Kaizuka T. Kishi C. Takamura A. Miura Y. (2009). Nutrient-dependent mTORC1 association with the ULK1-Atg13-FIP200 complex required for autophagy. *Mol. Biol. Cell* 20 1981–1991. 10.1091/mbc.E08 19211835 PMC2663915

[B59] Hsiao C. T. Tsai P. C. Lin C. C. Liu Y. T. Huang Y. H. Liao Y. C. (2016). Clinical and molecular characterization of BSCL2 mutations in a taiwanese cohort with hereditary neuropathy. *PLoS ONE* 11:e0147677 10.1371/journal.pone.0147677 PMC4729478 26815532

[B60] Huber N. Bieniossek C. Wagner K. M. Elsässer H.-P. Suter U. Berger I. (2016). Glutathione-conjugating and membrane-remodeling activity of GDAP1 relies on amphipathic C-terminal domain. *Sci. Rep.* 6:36930 10.1038/srep36930 PMC5107993 27841286

[B61] Huett A. Heath R. J. Begun J. Sassi S. O. Baxt L. A. Vyas J. M. (2012). The LRR and RING domain protein LRSAM1 is an E3 ligase crucial for ubiquitin-dependent autophagy of intracellular *Salmonella* typhimurium. *Cell Host Microbe* 12 778–790. 10.1016/j.chom.2012.10.019 23245322 PMC3785244

[B62] Ikenaka K. Kawai K. Katsuno M. Huang Z. Jiang Y. M. Iguchi Y. (2013). dnc-1/dynactin 1 knockdown disrupts transport of autophagosomes and induces motor neuron degeneration. *PLoS ONE* 8:e54511 10.1371/journal.pone.0054511 PMC3567092 23408943

[B63] Indo Y. Tsuruta M. Hayashida Y. Karim M. Ohta K. Kawano T. (1996). Mutations in the TRKA/NGF receptor gene in patients with congenital insensitivity to pain with anhidrosis. *Nat. Genet.* 13 485–488. 10.1038/ng1096-146 8696348

[B64] Irobi J. Van Impe K. Seeman P. Jordanova A. Dierick I. Verpoorten N. (2004). Hot-spot residue in small heat-shock protein 22 causes distal motor neuropathy. *Nat. Genet.* 36 597–601. 10.1038/ng1328 15122253

[B65] Ito D. Fujisawa T. Iida H. Suzuki N. (2008). Characterization of seipin/BSCL2 a protein associated with spastic paraplegia 17. *Neurobiol. Dis.* 31 266–277. 10.1016/j.nbd.2008.05.004 18585921

[B66] Jäger S. Bucci C. Tanida I. Ueno T. Kominami E. Saftig P. (2004). Role for Rab7 in maturation of late autophagic vacuoles. *J. Cell Sci.* 117 4837–4848. 10.1242/jcs.01370 15340014

[B67] Jang S. Y. Shin Y. K. Park S. Y. Park J. Y. Rha S. H. Kim J. K. (2015). Autophagy is involved in the reduction of myelinating schwann cell cytoplasm during myelin maturation of the peripheral nerve. *PLoS ONE* 10:e0116624 10.1371/journal.pone.0116624 PMC4291222 25581066

[B68] Jean S. Cox S. Nassari S. Kiger A. (2015). Starvation-induced MTMR 13 and RAB 21 activity regulates VAMP 8 to promote autophagosome – lysosome fusion. *EMBO Rep.* 16 297–311. 10.15252/embr.201439464 25648148 PMC4364869

[B69] Jiang P. Nishimura T. Sakamaki Y. Itakura E. Hatta T. Natsume T. (2014). The HOPS complex mediates autophagosome-lysosome fusion through interaction with syntaxin 17. *Mol. Biol. Cell* 25 1327–1337. 10.1091/mbc.E13-08-0447 24554770 PMC3982997

[B70] Ju J. S. Fuentealba R. A. Miller S. E. Jackson E. Piwnica-Worms D. Baloh R. H. (2009). Valosin-containing protein (VCP) is required for autophagy and is disrupted in VCP disease. *J. Cell Biol.* 187 875–888. 10.1083/jcb.200908115 20008565 PMC2806317

[B71] Kabeya Y. (2000). LC3 a mammalian homologue of yeast Apg8p, is localized in autophagosome membranes after processing. *EMBO J.* 19 5720–5728. 10.1093/emboj/19.21.5720 11060023 PMC305793

[B72] Kalaydjieva L. Gresham D. Gooding R. Heather L. Baas F. de Jonge R. (2000). N-myc downstream-regulated gene 1 is mutated in hereditary motor and sensory neuropathy-Lom. *Am. J. Hum. Genet.* 67 47–58. 10.1086/302978 10831399 PMC1287101

[B73] Katona I. Zhang X. Bai Y. Shy M. E. Guo J. Yan Q. (2011). Distinct pathogenic processes between Fig4-deficient motor and sensory neurons. *Eur. J. Neurosci.* 33 1401–1410. 10.1111/j.1460-9568.2011.07651.x 21410794

[B74] Khaminets A. Heinrich T. Mari M. Huebner A. K. Akutsu M. Grumati P. (2015). Regulation of endoplasmic reticulum turnover by FAM134B-mediated selective autophagy. *Nature* 522 354–358. 10.1038/nature14498 26040720

[B75] Kimonis V. E. Kovach M. J. Waggoner B. Leal S. Salam A. Rimer L. (2000). Clinical and molecular studies in a unique family with autosomal dominant limb-girdle muscular dystrophy and Paget disease of bone. *Genet. Med.* 2 232–241. 10.1097/00125817-200007000-00006 11252708 PMC6173187

[B76] Klionsky D. J. Abdelmohsen K. Abe A. Abedin M. J. Abeliovich H. Acevedo Arozena A. (2016). Guidelines for the use and interpretation of assays for monitoring autophagy (3rd edition). *Autophagy* 12 1–222. 10.1080/15548627.2015.1100356 26799652 PMC4835977

[B77] Komatsu M. Waguri S. Chiba T. Murata S. Iwata J. Tanida I. (2006). Loss of autophagy in the central nervous system causes neurodegeneration in mice. *Nature* 441 880–884. 10.1038/nature04723 16625205

[B78] Köttgen M. Buchholz B. Garcia-Gonzalez M. A. Kotsis F. Fu X. Doerken M. (2008). TRPP2 and TRPV4 form a polymodal sensory channel complex. *J. Cell Biol.* 182 437–447. 10.1083/jcb.200805124 18695040 PMC2500130

[B79] Kurth I. Pamminger T. Hennings J. C. Soehendra D. Huebner A. K. Rotthier A. (2009). Mutations in FAM134B, encoding a newly identified Golgi protein, cause severe sensory and autonomic neuropathy. *Nat. Genet.* 41 1179–1181. 10.1038/ng.464 19838196

[B80] Kwok A. S. Phadwal K. Turner B. J. Oliver P. L. Raw A. Simon A. K. (2011). HspB8 mutation causing hereditary distal motor neuropathy impairs lysosomal delivery of autophagosomes. *J. Neurochem.* 119 1155–1161. 10.1111/j.1471-4159.2011.07521.x 21985219

[B81] Kyöstilä K. Syrjä P. Jagannathan V. Chandrasekar G. Jokinen T. S. Seppälä E. H. (2015). A missense change in the ATG4D gene links aberrant autophagy to a neurodegenerative vacuolar storage disease. *PLoS Genet.* 11:e1005169 10.1371/journal.pgen.1005169 PMC4398399 25875846

[B82] Lacerda A. F. E. Hartjes E. Brunetti C. R. (2014). LITAF mutations associated with Charcot-Marie-Tooth disease 1C show mislocalization from the late endosome/lysosome to the mitochondria. *PLoS ONE* 9:e103454 10.1371/journal.pone.0103454 PMC4110028 25058650

[B83] Lamb C. A. Nühlen S. Judith D. Frith D. Snijders A. P. Behrends C. (2016). TBC1D14 regulates autophagy via the TRAPP complex and ATG9 traffic. *EMBO J.* 35 281–301. 10.15252/embj.201592695 26711178 PMC4741301

[B84] Larroquette F. Seto L. Gaub P. L. Kamal B. Wallis D. Larivière R. (2015). Vapb/Amyotrophic lateral sclerosis 8 knock-in mice display slowly progressive motor behavior defects accompanying ER stress and autophagic response. *Hum. Mol. Genet.* 24 6515–6529. 10.1093/hmg/ddv360 26362257 PMC4614709

[B85] Lee J.-Y. Koga H. Kawaguchi Y. Tang W. Wong E. Gao Y.-S. (2010). HDAC6 controls autophagosome maturation essential for ubiquitin-selective quality-control autophagy. *EMBO J.* 29 969–980. 10.1038/emboj.2009.405 20075865 PMC2837169

[B86] Lee S. M. Olzmann J. A. Chin L.-S. Li L. Babst M. Bendtsen J. D. (2011). Mutations associated with Charcot-Marie-Tooth disease cause SIMPLE protein mislocalization and degradation by the proteasome and aggresome-autophagy pathways. *J. Cell Sci.* 124 3319–3331. 10.1242/jcs.087114 21896645 PMC3178453

[B87] Lenk G. M. Meisler M. H. (2014). *Mouse Models of PI(35)P2 Deficiency with Impaired Lysosome Function* 1st Edn. Amsterdam: Elsevier Inc 10.1016/B978-0-12-397926-1.00014-7 PMC4059992 24359958

[B88] Lev S. Ben Halevy D. Peretti D. Dahan N. (2008). The VAP protein family: from cellular functions to motor neuron disease. *Trends Cell Biol.* 18 282–290. 10.1016/j.tcb.2008.03.006 18468439

[B89] Li J. Kim S. G. Blenis J. (2014). Rapamycin: one drug, many effects. *Cell Metab.* 19 373–379. 10.1016/j.cmet.2014.01.001 24508508 PMC3972801

[B90] Li J. Parker B. Martyn C. Natarajan C. Guo J. (2012). The PMP22 gene and its related diseases. *Mol. Neurobiol.* 47 673–698. 10.1007/s12035-012-8370-x 23224996 PMC3594637

[B91] Liang C. Lee J. Inn K. Gack M. U. Li Q. Roberts E. A. (2008). Beclin1-binding UVRAG targets the class C Vps complex to coordinate autophagosome maturation and endocytic trafficking. *Nat. Cell Biol.* 10 776–787. 10.1038/ncb1740 18552835 PMC2878716

[B92] Lipinski M. M. Zheng B. Lu T. Yan Z. Py B. F. Ng A. (2010). Genome-wide analysis reveals mechanisms modulating autophagy in normal brain aging and in Alzheimer’s disease. *Proc. Natl. Acad. Sci. U.S.A.* 107 14164–14169. 10.1073/pnas.1009485107 20660724 PMC2922576

[B93] Liu J. Xing H. Chen Y. Wang L. Wang D. Rao Q. (2012). PIG7 transactivated by AML1 promotes apoptosis and differentiation of leukemia cells with AML1-ETO fusion gene. *Leukemia* 26 117–126. 10.1038/leu.2011.178 21836606

[B94] Lo K. Y. Kuzmin A. Unger S. M. Petersen J. D. Silverman M. A. (2011). KIF1A is the primary anterograde motor protein required for the axonal transport of dense-core vesicles in cultured hippocampal neurons. *Neurosci. Lett.* 491 168–173. 10.1016/j.neulet.2011.01.018 21256924

[B95] Longatti A. Lamb C. A. Razi M. Yoshimura S. I. Barr F. A. Tooze S. A. (2012). TBC1D14 regulates autophagosome formation via Rab11- and ULK1-positive recycling endosomes. *J. Cell Biol.* 197 659–675. 10.1083/jcb.201111079 22613832 PMC3365497

[B96] Lu J. H. Tan J. Q. Durairajan S. S. K. Liu L. F. Zhang Z. H. Ma L. (2012). Isorhynchophylline, a natural alkaloid, promotes the degradation of α-synuclein in neuronal cells via inducing autophagy. *Autophagy* 8 98–108. 10.4161/auto.8.1.18313 22113202

[B97] Lupski J. R. Wise C. A. Kuwano A. Pentao L. Parke J. T. Glaze D. G. (1992). Gene dosage is a mechanism for Charcot-Marie-Tooth disease type 1A. *Nat. Genet.* 1 29–33. 10.1038/ng0492-29 1301995

[B98] Madorsky I. Opalach K. Waber A. Verrier J. D. Solmo C. Foster T. (2009). Intermittent fasting alleviates the neuropathic phenotype in a mouse model of Charcot-Marie-Tooth disease. *Neurobiol. Dis.* 34 146–154. 10.1016/j.nbd.2009.01.002 19320048 PMC2757933

[B99] Mari M. Griffith J. Rieter E. Krishnappa L. Klionsky D. J. Reggiori F. (2010). An Atg9-containing compartment that functions in the early steps of autophagosome biogenesis. *J. Cell Biol.* 190 1005–1022. 10.1083/jcb.200912089 20855505 PMC3101592

[B100] McNeill A. Magalhaes J. Shen C. Chau K. Y. Hughes D. Mehta A. (2014). Ambroxol improves lysosomal biochemistry in glucocerebrosidase mutation-linked Parkinson disease cells. *Brain* 137 1481–1495. 10.1093/brain/awu020 24574503 PMC3999713

[B101] Mersiyanova I. V. Perepelov A. V. Polyakov A. V. Sitnikov V. F. Dadali E. L. Oparin R. B. (2000). A new variant of Charcot-Marie-Tooth disease type 2 is probably the result of a mutation in the neurofilament-light gene. *Am. J. Hum. Genet.* 67 37–46. 10.1086/302962 10841809 PMC1287099

[B102] Mijaljica D. Prescott M. Devenish R. J. (2011). Microautophagy in mammalian cells: revisiting a 40-year-old conundrum. *Autophagy* 7 673–682. 10.4161/auto.7.7.14733 21646866

[B103] Miller F. D. Kaplan D. R. (2001). Neurotrophin signalling pathways regulating neuronal apoptosis. *Cell. Mol. Life Sci.* 58 1045–1053. 11529497 10.1007/PL00000919 PMC11337384

[B104] Moriguchi T. Urushiyama S. Hisamoto N. Iemura S. I. Uchida S. Natsume T. (2005). WNK1 regulates phosphorylation of cation-chloride-coupled cotransporters via the STE20-related kinases. SPAK and OSR1. *J. Biol. Chem.* 280 42685–42693. 10.1074/jbc.M510042200 16263722

[B105] Murrow L. Malhotra R. Debnath J. (2015). ATG12–ATG3 interacts with Alix to promote basal autophagic flux and late endosome function. *Nat. Cell Biol.* 17 300–310. 10.1038/ncb3112 25686249 PMC4344874

[B106] Nalbandian A. Llewellyn K. J. Badadani M. Yin H. Z. Nguyen C. Katheria V. (2013). A progressive translational mouse model of human valosin-containing protein disease: the VCPR155H/+ mouse. *Muscle Nerve* 47 260–270. 10.1002/mus.23522 23169451 PMC3556223

[B107] Ng A. C. Y. Eisenberg J. M. Heath R. J. W. Huett A. Robinson C. M. Nau G. J. (2011). Human leucine-rich repeat proteins: a genome-wide bioinformatic categorization and functional analysis in innate immunity. *Proc. Natl. Acad. Sci. U.S.A.* 108(Suppl. 1) 4631–4638. 10.1073/pnas.1000093107 20616063 PMC3063585

[B108] Nicholson G. A. Valentijn L. J. Cherryson A. K. Kennerson M. L. Bragg T. L. DeKroon R. M. (1994). A frame shift mutation in the PMP22 gene in hereditary neuropathy with liability to pressure palsies. *Nat. Genet.* 6 263–266. 10.1038/ng0394-263 8012388

[B109] Niemann A. Ruegg M. La Padula V. Schenone A. Suter U. (2005). Ganglioside-induced differentiation associated protein 1 is a regulator of the mitochondrial network: new implications for Charcot-Marie-Tooth disease. *J. Cell Biol.* 170 1067–1078. 10.1083/jcb.200507087 16172208 PMC2171517

[B110] Nishimura A. L. Mitne-Neto M. Silva H. C. A. Richieri-Costa A. Middleton S. Cascio D. (2004). A mutation in the vesicle-trafficking protein VAPB causes late-onset spinal muscular atrophy and amyotrophic lateral sclerosis. *Am. J. Hum. Genet.* 75 822–831. 10.1086/425287 15372378 PMC1182111

[B111] Notterpek L. Shooter E. M. Snipes G. J. (1997). Upregulation of the endosomal-lysosomal pathway in the trembler-J neuropathy. *J. Neurosci.* 17 4190–4200. 9151736 10.1523/JNEUROSCI.17-11-04190.1997 PMC6573524

[B112] Novoselov S. S. Mustill W. J. Gray A. L. Dick J. R. Kanuga N. Kalmar B. (2013). Molecular chaperone mediated late-stage neuroprotection in the SOD1G93A mouse model of amyotrophic lateral sclerosis. *PLoS ONE* 8:e73944 10.1371/journal.pone.0073944 PMC3758296 24023695

[B113] Ohta K. Mizuno A. Ueda M. Li S. Suzuki Y. Hida Y. (2010). Autophagy impairment stimulates PS1 expression and γ-secretase activity. *Autophagy* 6 345–352. 10.4161/auto.6.3.11228 20168091

[B114] Oz-Levi D. Ben-Zeev B. Ruzzo E. K. Hitomi Y. Gelman A. Pelak K. (2012). Mutation in TECPR2 reveals a role for autophagy in hereditary spastic paraparesis. *Am. J. Hum. Genet.* 91 1065–1072. 10.1016/j.ajhg.2012.09.015 23176824 PMC3516605

[B115] Papadopoulos C. Kirchner P. Bug M. Grum D. Koerver L. Schulze N. (2016). VCP/p97 cooperates with YOD1 UBXD1 and PLAA to drive clearance of ruptured lysosomes by autophagy. *EMBO J.* 36 135–150. 10.15252/embj.201695148 27753622 PMC5242375

[B116] Pareyson D. Saveri P. Piscosquito G. (2014). Charcot-Marie-Tooth disease and related hereditary neuropathies : from gene function to associated phenotypes. *Curr. Mol. Med.* 14 1009–1033. 10.2174/1566524014666141010154205 25323870

[B117] Parzych K. R. Klionsky D. J. (2014). An overview of autophagy: morphology, mechanism, and regulation. *Antioxid. Redox Signal.* 20 460–473. 10.1089/ars.2013.5371 23725295 PMC3894687

[B118] Pattingre S. Tassa A. Qu X. Garuti R. Xiao H. L. Mizushima N. (2005). Bcl-2 antiapoptotic proteins inhibit Beclin 1-dependent autophagy. *Cell* 122 927–939. 10.1016/j.cell.2005.07.002 16179260

[B119] Pavel M. Imarisio S. Menzies F. M. Jimenez-Sanchez M. Siddiqi F. H. Wu X. (2016). CCT complex restricts neuropathogenic protein aggregation via autophagy. *Nat. Commun.* 7:13821 10.1038/ncomms13821 PMC5155164 27929117

[B120] Perrot R. Berges R. Bocquet A. Eyer J. (2008). Review of the multiple aspects of neurofilament functions, and their possible contribution to neurodegeneration. *Mol. Neurobiol.* 38 27–65. 10.1007/s12035-008-8033-0 18649148

[B121] Petiot A. Ogier-denis E. Edward F. C. Meijer A. J. Chem J. B. Blommaart E. F. C. (2000). Distinct classes of phosphatidylinositol 3’-kinases are involved in signaling pathways that control macroautophagy in HT-29 cells. *J. Biol. Chem.* 275 992–998. 10.1074/jbc.275.2.992 10625637

[B122] Previtali S. C. Zerega B. Sherman D. L. Brophy P. J. Dina G. King R. H. M. (2003). Myotubularin-related 2 protein phosphatase and neurofilament light chain protein, both mutated in CMT neuropathies, interact in peripheral nerve. *Hum. Mol. Genet.* 12 1713–1723. 10.1093/hmg/ddg179 12837694

[B123] Prior R. Van Helleputte L. Benoy V. Den Bosch L. Van (2017). Defective axonal transport: a common pathological mechanism in inherited and acquired peripheral neuropathies. *Neurobiol. Dis.* 10.1016/j.nbd.2017.02.009 [Epub ahead of print]. 28238949

[B124] Puls I. Jonnakuty C. LaMonte B. H. Holzbaur E. L. F. Tokito M. Mann E. (2003). Mutant dynactin in motor neuron disease. *Nat. Genet.* 33 455–456. 10.1038/ng1123 12627231

[B125] Rahvar M. Nikseresht M. Shafiee S. M. Naghibalhossaini F. Rasti M. Panjehshahin M. R. (2011). Effect of oral resveratrol on the BDNF gene expression in the hippocampus of the rat brain. *Neurochem. Res.* 36 761–765. 10.1007/s11064-010-0396-398 21221775

[B126] Rangaraju S. Verrier J. D. Madorsky I. Nicks J. Dunn W. A. Notterpek L. (2010). Rapamycin activates autophagy and improves myelination in explant cultures from neuropathic mice. *J. Neurosci.* 30 11388–11397. 10.1523/JNEUROSCI.1356-10.2010 20739560 PMC3478092

[B127] Rivière J. B. Verlaan D. J. Shekarabi M. Lafrenière R. G. Bénard M. Der Kaloustian V. M. (2004). A mutation in the *HSN2* gene causes sensory neuropathy type II in a lebanese family. *Ann. Neurol.* 56 572–575. 10.1002/ana.20237 15455397

[B128] Rivire J. B. Ramalingam S. Lavastre V. Shekarabi M. Holbert S. Lafontaine J. (2011). KIF1A, an axonal transporter of synaptic vesicles, is mutated in hereditary sensory and autonomic neuropathy type 2. *Am. J. Hum. Genet.* 89 219–301. 10.1016/j.ajhg.2011.06.013 21820098 PMC3155159

[B129] Rizzo F. Ronchi D. Salani S. Nizzardo M. Fortunato F. Bordoni A. (2016). Selective mitochondrial depletion, apoptosis resistance, and increased mitophagy in human Charcot-Marie-Tooth 2A motor neurons. *Hum. Mol. Genet.* 25 4266–4281. 10.1093/hmg/ddw258 27506976

[B130] Roberts R. C. Peden A. A. Buss F. Bright N. A. Latouche M. Reilly M. M. (2010). Mistargeting of SH3TC2 away from the recycling endosome causes Charcot-Marie-Tooth disease type 4C. *Hum. Mol. Genet.* 19 1009–1018. 10.1093/hmg/ddp565 20028792 PMC2830826

[B131] Roberts-Clarke D. Fornusek C. Saigal N. Halaki M. Burns J. Nicholson G. (2016). Relationship between physical performance and quality of life in Charcot-Marie-Tooth disease: a pilot study. *J. Peripher. Nerv. Syst.* 21 357–364. 10.1111/jns.12191 27699915

[B132] Robinson F. L. Dixon J. E. (2005). The phosphoinositide-3-phosphatase MTMR2 associates with MTMR13 a membrane-associated pseudophosphatase also mutated in type 4B Charcot-Marie-Tooth disease. *J. Biol. Chem.* 280 31699–31707. 10.1074/jbc.M505159200 15998640

[B133] Rose C. Menzies F. M. Renna M. Acevedo-Arozena A. Corrochano S. Sadiq O. (2010). Rilmenidine attenuates toxicity of polyglutamine expansions in a mouse model of Huntington’s disease. *Hum. Mol. Genet.* 19 2144–2153. 10.1093/hmg/ddq093 20190273 PMC2865373

[B134] Rose J. M. Novoselov S. S. Robinson P. A. Cheetham M. E. (2011). Molecular chaperone-mediated rescue of mitophagy by a Parkin RING1 domain mutant. *Hum. Mol. Genet.* 20 16–27. 10.1093/hmg/ddq428 20889486 PMC3000674

[B135] Rossor A. M. Kalmar B. Greensmith L. Reilly M. M. (2012). The distal hereditary motor neuropathies. *J. Neurol. Neurosurg. Psychiatry* 83 6–14. 10.1136/jnnp-2011-300952 22028385

[B136] Rotthier A. Baets J. Timmerman V. Janssens K. (2012). Mechanisms of disease in hereditary sensory and autonomic neuropathies. *Nat. Rev. Neurol.* 8 73–85. 10.1038/nrneurol.2011.227 22270030

[B137] Russo M. Laurá M. Polke J. M. Davis M. B. Blake J. Brandner S. (2011). Variable phenotypes are associated with PMP22 missense mutations. *Neuromuscul. Disord.* 21 106–114. 10.1016/j.nmd.2010.11.011 21194947

[B138] Sahni S. Bae D. H. Lane D. J. R. Kovacevic Z. Kalinowski D. S. Jansson P. J. (2014). The metastasis suppressor, N-myc Downstream-regulated gene 1 (NDRG1), inhibits stress-induced autophagy in cancer cells. *J. Biol. Chem.* 289 9692–9709. 10.1074/jbc.M113.529511 24532803 PMC3975018

[B139] Saitsu H. Nishimura T. Muramatsu K. Kodera H. Kumada S. Sugai K. (2013). De novo mutations in the autophagy gene WDR45 cause static encephalopathy of childhood with neurodegeneration in adulthood. *Nat. Genet.* 45 445–449. 10.1038/ng.2562 23435086

[B140] Sanchez E. Darvish H. Mesias R. Taghavi S. Firouzabadi S. G. Walker R. H. (2016). Identification of a large DNAJB2 deletion in a family with spinal muscular atrophy and parkinsonism. *Hum. Mutat.* 37 1180–1189. 10.1002/humu.23055 27449489 PMC5375037

[B141] Santel A. Fuller M. T. (2001). Control of mitochondrial morphology by a human mitofusin. *J. Cell Sci.* 114 867–874. 11181170 10.1242/jcs.114.5.867

[B142] Sarkar S. Davies J. E. Huang Z. Tunnacliffe A. Rubinsztein D. C. (2007). Trehalose, a novel mTOR-independent autophagy enhancer, accelerates the clearance of mutant huntingtin and α-synuclein. *J. Biol. Chem.* 282 5641–5652. 10.1074/jbc.M609532200 17182613

[B143] Sasaki T. Gotow T. Shiozaki M. Sakaue F. Saito T. Julien J. P. (2006). Aggregate formation and phosphorylation of neurofilament-L Pro22 Charcot-Marie-Tooth disease mutants. *Hum. Mol. Genet.* 15 943–952. 10.1093/hmg/ddl011 16452125

[B144] Schrepfer E. Scorrano L. (2016). Mitofusins, from mitochondria to metabolism. *Mol. Cell* 61 683–694. 10.1016/j.molcel.2016.02.022 26942673

[B145] Schroer T. A. (2004). Dynactin. *Annu. Rev. Cell Dev. Biol.* 20 759–779. 10.1146/annurev.cellbio.20.012103.094623 15473859

[B146] Schulze R. J. McNiven M. A. (2014). A well-oiled machine DNM2/dynamin 2 helps keep hepatocyte lipophagy running smoothly. *Autophagy* 10 388–389. 10.4161/auto.27486 24351653 PMC4028321

[B147] Sebastián D. Sorianello E. Segalés J. Irazoki A. Ruiz-Bonilla V. Sala D. (2016). Mfn2 deficiency links age-related sarcopenia and impaired autophagy to activation of an adaptive mitophagy pathway. *EMBO J.* 35 1677–1693. 10.15252/embj.201593084 27334614 PMC4969577

[B148] Senderek J. Bergmann C. Ramaekers V. T. Nelis E. Bernert G. Makowski A. (2003a). Mutations in the ganglioside-induced differentiation-associated protein-1 (GDAP1) gene in intermediate type autosomal recessive Charcot-Marie-Tooth neuropathy. *Brain* 126 642–649. 10.1093/brain/awg068 12566285

[B149] Senderek J. Bergmann C. Stendel C. Kirfel J. Verpoorten N. De Jonghe P. (2003b). Mutations in a gene encoding a novel SH3/TPR domain protein cause autosomal recessive Charcot-Marie-Tooth type 4C neuropathy. *Am. J. Hum. Genet.* 73 1106–1119. 10.1086/379525 14574644 PMC1180490

[B150] Shaw C. E. (2010). Capturing VCP: another molecular piece in the ALS jigsaw puzzle. *Neuron* 68 812–814. 10.1016/j.neuron.2010.11.040 21144996

[B151] Shintani T. Mizushima N. Ogawa Y. Matsuura A. Noda T. Ohsumi Y. (1999). Apg10p, a novel protein-conjugating enzyme essential for autophagy in yeast. *EMBO J.* 18 5234–5241. 10.1093/emboj/18.19.5234 10508157 PMC1171594

[B152] Sivera R. Espinõs C. Vílchez J. J. Mas F. Martínez-Rubio D. Chumillas M. J. (2010). Phenotypical features of the p.R120W mutation in the GDAP1 gene causing autosomal dominant Charcot-Marie-Tooth disease. *J. Peripher. Nerv. Syst.* 15 334–344. 10.1111/j.1529-8027.2010.00286.x 21199105

[B153] Spencer B. Potkar R. Trejo M. Rockenstein E. Patrick C. Gindi R. (2009). Beclin 1 gene transfer activates autophagy and ameliorates the neurodegenerative pathology in alpha-synuclein models of Parkinson’s and Lewy body diseases. *J. Neurosci.* 29 13578–13588. 10.1523/JNEUROSCI.4390-09.2009 19864570 PMC2812014

[B154] Stadel D. Millarte V. Tillmann K. D. Huber J. Tamin-Yecheskel B. C. Akutsu M. (2015). TECPR2 cooperates with LC3C to regulate COPII-dependent ER export. *Mol. Cell* 60 89–104. 10.1016/j.molcel.2015.09.010 26431026

[B155] Stavoe A. K. H. Hill S. E. Hall D. H. Colón-Ramos D. A. (2016). KIF1A/UNC-104 transports ATG-9 to regulate neurodevelopment and autophagy at synapses. *Dev. Cell* 38 171–185. 10.1016/j.devcel.2016.06.012 27396362 PMC4961624

[B156] Stendel C. Roos A. Kleine H. Arnaud E. Ozcelik M. Sidiropoulos P. N. M. (2010). SH3TC2 a protein mutant in Charcot-Marie-Tooth neuropathy, links peripheral nerve myelination to endosomal recycling. *Brain* 133 2462–2474. 10.1093/brain/awq168 20826437

[B157] Stenmark H. (2009). Rab GTPases as coordinators of vesicle traffic. *Nat. Rev. Mol. Cell Biol.* 10 513–525. 10.1038/nrm2728 19603039

[B158] Stephan J. S. Yeh Y.-Y. Ramachandran V. Deminoff S. J. Herman P. K. (2009). The Tor and PKA signaling pathways independently target the Atg1/Atg13 protein kinase complex to control autophagy. *Proc. Natl. Acad. Sci. U.S.A.* 106 17049–17054. 10.1073/pnas.0903316106 19805182 PMC2761351

[B159] Stojkovic T. (2016). Hereditary neuropathies: an update. *Rev. Neurol.* 172 775–778. 10.1016/j.neurol.2016.06.007 27866730

[B160] Street V. A. Bennett C. L. Goldy J. D. Shirk A. J. Kleopa K. A. Tempel B. L. (2003). Mutation of a putative protein degradation gene LITAF/SIMPLE in Charcot-Marie-Tooth disease 1C. *Neurology* 60 22–26. 10.1212/WNL.60.1.22 12525712

[B161] Takahashi Y. Tsotakos N. Liu Y. Young M. M. Serfass J. Tang Z. (2016). The Bif-1-Dynamin 2 membrane fission machinery regulates Atg9-containing vesicle generation at the Rab11-positive reservoirs. *Oncotarget* 7 20855–20868. 10.18632/oncotarget.8028 26980706 PMC4991497

[B162] Tang B. S. Zhao G. H. Luo W. Xia K. Cai F. Pan Q. (2005). Small heat-shock protein 22 mutated in autosomal dominant Charcot-Marie-Tooth disease type 2L. *Hum. Genet.* 116 222–224. 15565283 10.1007/s00439-004-1218-3

[B163] Tang X. Metzger D. Leeman S. Amar S. (2006). LPS-induced TNF-α factor (LITAF)-deficient mice express reduced LPS-induced cytokine: evidence for LITAF-dependent LPS signaling pathways. *Proc. Natl. Acad. Sci. U.S.A.* 103 13777–13782. 10.1073/pnas.0605988103 16954198 PMC1560089

[B164] Tanida I. Mizushima N. Kiyooka M. Ohsumi M. Ueno T. Ohsumi Y. (1999). Apg7p/Cvt2p: a novel protein-activating enzyme essential for autophagy. *Mol. Biol. Cell* 10 1367–1379. 10.1091/mbc.10.5.1367 10233150 PMC25280

[B165] Thomas P. D. P. K. (2005). *Peripheral Neuropathy.* Available at: https://www.elsevier.com/books/peripheral-neuropathy/dyck/978-0-7216-9491-7

[B166] Timmerman V. Nelis E. Van Hul W. Nieuwenhuijsen B. W. Chen K. L. Wang S. (1992). The peripheral myelin protein gene PMP-22 is contained within the Charcot-Marie-Tooth disease type 1A duplication. *Nat. Genet.* 1 171–175. 10.1038/ng0692-171 1303230

[B167] Timmerman V. Strickland A. V. Züchner S. (2014). Genetics of Charcot-Marie-Tooth (CMT) disease within the frame of the human genome project success. *Genes* 5 13–32. 10.3390/genes5010013 24705285 PMC3978509

[B168] Towers C. G. Thorburn A. (2016). Therapeutic targeting of autophagy. *EBioMedicine* 14 15–23. 10.1016/j.ebiom.2016.10.034 28029600 PMC5161418

[B169] Vaccari I. Carbone A. Previtali S. C. Mironova Y. A. Alberizzi V. Noseda R. (2015). Loss of Fig4 in both Schwann cells and motor neurons contributes to CMT4J neuropathy. *Hum. Mol. Genet.* 24 383–396. 10.1093/hmg/ddu451 25187576 PMC4275070

[B170] Vallat J. M. Goizet C. Tazir M. Couratier P. Magy L. Mathis S. (2016). Classifications of neurogenetic diseases: an increasingly complex problem. *Rev. Neurol.* 172 339–349. 10.1016/j.neurol.2016.04.005 27240993

[B171] Verhoeven K. De Jonghe P. Coen K. Verpoorten N. Auer-Grumbach M. Kwon J. M. (2003). Mutations in the small GTP-ase late endosomal protein RAB7 cause Charcot-Marie-Tooth type 2B neuropathy. *Am. J. Hum. Genet.* 72 722–727. 10.1086/367847 12545426 PMC1180247

[B172] Vingtdeux V. Giliberto L. Zhao H. Chandakkar P. Wu Q. Simon J. E. (2010). AMP-activated protein kinase signaling activation by resveratrol modulates amyloid-β peptide metabolism. *J. Biol. Chem.* 285 9100–9113. 10.1074/jbc.M109.060061 20080969 PMC2838330

[B173] Vita G. La Foresta S. Russo M. Vita G. L. Messina S. Lunetta C. (2016). Sport activity in Charcot-Marie-Tooth disease: a case study of a Paralympic swimmer. *Neuromuscul. Disord.* 26 614–618. 10.1016/j.nmd.2016.06.002 27460291 PMC5026044

[B174] Wang Z. Hu J. Li G. Qu L. He Q. Lou Y. (2014). PHF23 (plant homeodomain finger protein 23) negatively regulates cell autophagy by promoting ubiquitination and degradation of E3 ligase LRSAM1. *Autophagy* 10 2158–2170. 10.4161/auto.36439 25484098 PMC4502667

[B175] Weis J. Claeys K. G. Roos A. Azzedine H. Katona I. Schroder J. M. (2016). Towards a functional pathology of hereditary neuropathies. *Acta Neuropathol.* 133 493–515. 10.1007/s00401-016-1645-y 27896434

[B176] Westhoff B. Chapple J. P. Van Der Spuy J. Hohfeld J. Cheetham M. E. (2005). HSJ1 is a neuronal shuttling factor for the sorting of chaperone clients to the proteasome. *Curr. Biol.* 15 1058–1064. 10.1016/j.cub.2005.04.058 15936278

[B177] Weterman M. A. J. Sorrentino V. Kasher P. R. Jakobs M. E. van Engelen B. G. M. Fluiter K. (2012). A frameshift mutation in LRSAM1 is responsible for a dominant hereditary polyneuropathy. *Hum. Mol. Genet.* 21 358–370. 10.1093/hmg/ddr471 22012984 PMC3276280

[B178] Wiesner D. Sinniger J. Henriques A. Dieterlé S. Müller H. P. Rasche V. (2015). Low dietary protein content alleviates motor symptoms in mice with mutant dynactin/dynein-mediated neurodegeneration. *Hum. Mol. Genet.* 24 2228–2240. 10.1093/hmg/ddu741 25552654 PMC4447824

[B179] Windpassinger C. Auer-Grumbach M. Irobi J. Patel H. Petek E. Hörl G. (2004). Heterozygous missense mutations in BSCL2 are associated with distal hereditary motor neuropathy and Silver syndrome. *Nat. Genet.* 36 271–276. 10.1038/ng1313 14981520

[B180] Yang Z. Klionsky D. J. (2010). Mammalian autophagy: core molecular machinery and signaling regulation. *Curr. Opin. Cell Biol.* 22 124–131. 10.1016/j.ceb.2009.11.014 20034776 PMC2854249

[B181] Zhan L. Yang Y. Ma T. T. Huang C. Meng X. M. Zhang L. (2015). Transient receptor potential vanilloid 4 inhibits rat HSC-T6 apoptosis through induction of autophagy. *Mol. Cell. Biochem.* 402 9–22. 10.1007/s11010-014-2298-6 25600591

[B182] Zhou J. Yang Z. Tsuji T. Gong J. Xie J. Chen C. (2011). LITAF and TNFSF15 two downstream targets of AMPK, exert inhibitory effects on tumor growth. *Oncogene* 30 1892–1900. 10.1038/onc.2010.575 21217782 PMC3431012

[B183] Züchner S. Mersiyanova I. V. Muglia M. Bissar-Tadmouri N. Rochelle J. Dadali E. L. (2004). Mutations in the mitochondrial GTPase mitofusin 2 cause Charcot-Marie-Tooth neuropathy type 2A. *Nat. Genet.* 36 449–451. 10.1038/ng1341 15064763

[B184] Züchner S. Noureddine M. Kennerson M. Verhoeven K. Claeys K. De Jonghe P. (2005). Mutations in the pleckstrin homology domain of dynamin 2 cause dominant intermediate Charcot-Marie-Tooth disease. *Nat. Genet.* 37 289–294. 10.1212/WNL.64.10.1826-a 15731758

